# Theory for a non-invasive diagnostic biomarker for craniospinal diseases

**DOI:** 10.1016/j.nicl.2022.103280

**Published:** 2022-12-08

**Authors:** Fariba Karimi, Esra Neufeld, Arya Fallahi, Andrea Boraschi, Jaco J.M. Zwanenburg, Andreas Spiegelberg, Vartan Kurtcuoglu, Niels Kuster

**Affiliations:** a Foundation for Research on Information Technologies in Society (IT’IS), Zurich, Switzerland; b Department of Information Technology and Electrical Engineering, Swiss Federal Institute of Technology (ETH), Zurich, Switzerland; c The Interface Group, Institute of Physiology, University of Zurich, Zurich, Switzerland; d Zurich Center for Integrative Human Physiology, University of Zurich, Zurich, Switzerland; e Center for Image Sciences, University Medical Center Utrecht, Utrecht, The Netherlands

**Keywords:** Intracranial pressure, Craniospinal compliance, Non-invasive diagnostics, Craniospinal disease screening, Continuous monitoring, Computational electrostatics

## Abstract

Monitoring intracranial pressure (ICP) and craniospinal compliance (CC) is frequently required in the treatment of patients suffering from craniospinal diseases. However, current approaches are invasive and cannot provide continuous monitoring of CC. Dynamic exchange of blood and cerebrospinal fluid (CSF) between cranial and spinal compartments due to cardiac action transiently modulates the geometry and dielectric properties of the brain. The resulting impedance changes can be measured and might be usable as a non-invasive CC surrogate. A numerically robust and computationally efficient approach based on the reciprocity theorem was developed to compute dynamic impedance changes resulting from small geometry and material property changes. The approach was successfully verified against semi-analytical benchmarks, before being combined with experimental brain pulsation data to study the information content of the impedance variation. The results indicate that the measurable signal is dominated by the pulsatile displacement of the cortical brain surface, with minor contributions from the ventricular surfaces and from changes in brain perfusion. Different electrode setups result in complementary information. The information content from the investigated three electrode pairs was employed to successfully infer subject-specific brain pulsation and motion features. This suggests that non-invasive CC surrogates based on impedance monitoring could be established.

## Highlights

• Accurate and efficient modeling method for studying dynamic electromagnetic problems.
• Investigation of brain-pulsation-related, measurable head impedance variations.
• Application to 4D image data from healthy subjects to study inter-subject variability.
• Identification of relationships between measurable signals and pulsation features.
• Revelation of potential as non-invasive craniospinal compliance monitoring tool.

## Graphical abstract

**Figure:**
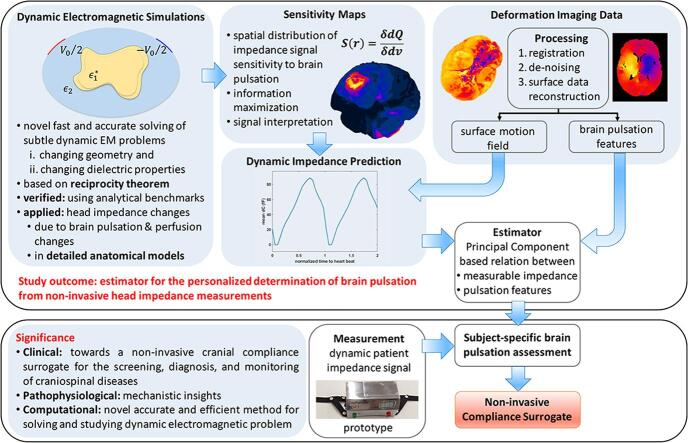

## 1 Introduction

The regulation of intracranial pressure (ICP) is vital for normal brain function. Several pathological conditions and neurological disorders including hydrocephalus, traumatic brain injury, stroke, and brain tumor can cause an increase in ICP beyond safe physiological margins. Due to the potentially severe pathological consequences of raised ICP, avoiding it is of utmost importance. Consequently, continuous monitoring and regulation of ICP, or of the underlying craniospinal compliance (CC), can be crucial for diagnosis and therapy. By CC we understand the sum of intracranial and spinal compliance; it is defined as the derivative $\frac{\mathrm{dV}}{d\mathrm{ICP}}$, where *V* is the cerebrospinal fluid (CSF) volume. The development of accurate, cost-effective, and non-invasive ICP/CC monitoring is therefore the focus of extensive research efforts (Zhang et al., 2017, Evensen and Eide, 2020).

Currently, there are different approaches for measuring ICP, which vary in invasiveness. The use of invasive, fluid-filled ventricular catheters connected to an external pressure sensor remains the gold standard for ICP monitoring (Foundation et al., 2000, Spiegelberg et al., 2015). However, this method has several drawbacks including high infection rates, postprocedural hemorrhaging, and mispositioning of catheters (Tavakoli et al., 2017). To resolve these problems, microsensors, such as the Spiegelberg ICP sensor (Whittle, 2000), have been developed in the early 1990s. While they are less-invasive than fluid-filled ventricular catheters (Whittle, 2000, Bekar et al., 2009), microsensors still suffer from shortcomings such as the risk of bleeding and infection (Evensen and Eide, 2020). The most important challenge in invasive methods is the need for surgical penetration of the skull and dura mater, which prevents application outside the intensive care unit (ICU) or for prolonged periods. In addition to ICP monitoring, CC assessment requires volume manipulation procedures using infusion testing (Bottan et al., 2013). Even though the infusion testing procedure has improved over time, it remains invasive and unsuitable for continuous CC monitoring (Jan Malm et al., 2012).

Hence, there is a great need to develop non-invasive approaches (Zhang et al., 2017). Non-invasive methods typically rely on measuring surrogate quantities that strongly correlate with ICP or CC (Zhang et al., 2017). These methods can be categorized according to their underlying working principle, including imaging of fluid-motion by magnetic resonance imaging (MRI) or transcranial Doppler ultrasonography (TCD), measurement of structural changes, and characterization of electric or acoustic properties (Zhang et al., 2017). MRI has been proposed as a tool for measuring ICP non-invasively (Alperin et al., 2000). Drawbacks of this method include high cost and unsuitability for long-term monitoring (Nag et al., 2019, Zhang et al., 2017). Ultrasound-based methods are much cheaper, but highly sensitive to the probe direction and patient anatomy (Zhang et al., 2017, Evensen and Eide, 2020).

As a result of cardiovascular and respiratory activity, blood and CSF are dynamically exchanged between cranial and spinal compartments, modulating ICP and being necessarily associated with variations in both head anatomical geometry and the dielectric property distributions, also under healthy physiologic conditions. Electrical impedance tomography (EIT) thus could be used to derive information about ICP. Manwaring et al. were the first to employ this method for continuous non-invasive ICP monitoring in animal models (Manwaring et al., 2013). Electric capacitance tomography (ECT), based on the same principle as EIT, has been applied to detect brain changes caused, for instance, by tumor growth (Taruno et al., 2013). Deriving an ICP surrogate from head capacitance measurements using electrically isolated electrodes on the scalp was proposed by Russegger et al. (1990) in the early 1990s. The head capacitance is small (∼100pF) and the changes that need to be accurately measured are less than 1% thereof. At the time, however, there was a lack of suitable hardware and computational algorithms with sufficient accuracy to measure these small changes.

Given the problems and limitation inherent in current ICP and CC measurement and monitoring modalities, it is clear that reliable non-invasive alternatives should be developed. The present work aims to support this effort by developing numerical techniques to computationally determine and investigate proposed head-impedance-based measurement approaches using image-based and highly detailed computational models. *In silico* methodologies can then be applied to investigate the relationships between measurable impedance signals and underlying dynamic anatomical and dielectric changes. As mentioned above, one of the key challenges results from the very small changes in magnitude of the head impedance – where ‘small’ refers to the magnitude of the impedance change that demands sensitive measurement equipment, and also to its relative magnitude compared to that of the total head impedance. This complicates both computations and experimental measurements. To overcome the measurement problems, Andreas Spiegelberg et al. (2022) developed a new non-invasive impedance measurement device. This work aims at overcoming the computational issues. Extracting small differences from simulations with their associated numerical errors (e.g., discretization- and convergence-related) is problematic. Another issue stems from the high computational effort that is amplified by the need to sufficiently resolve the dynamic changes in time. Corresponding novel computational methodologies to overcome these issues are developed and verified in this study. They make use of the reciprocity theorem and enable numerically robust and computationally efficient, highly detailed and realistic, personalized simulations of CC-related impedance-changes. Those methodologies are applicable well beyond the current application-of-interest, whenever the electromagnetic (EM) impact on impedance of small dynamic changes in geometry or dielectric properties are to be accurately assessed. In life sciences, this includes neural-activity or perfusion-related tissue impedance changes, as well as cardiovascular, respiratory, and digestive motion. While such changes can be negligible in many applications, they are increasingly important in various fields involving high-precision measurements, such as imaging, or neuroscientific diagnosis and therapy (e.g., Fultz et al., 2019).

The goals of this study are to:

• develop theory and methodology to efficiently and robustly compute small impedance changes resulting from (transient) changes in geometry and/or dielectric properties;
• validate the methodology and verify its implementation;
• derive a spatial sensitivity concept that can be used to understand the regional contribution to the measurable signal, e.g., for signal information content maximization;
• apply the methodology using a detailed anatomical head model and image-based 4D deformation data;
• investigate inter-subject differences in the obtainable signal and relate them to differences in brain pulsation features;
• investigate the information content of the measurable signal – also considering multiple electrode placements – in view of non-invasive, patient-specific brain pulsation inference, towards deriving a non-invasive CC surrogate; and
• discuss modeling uncertainties and limitations.

## 2 Methods

### 2.1 Theory

Exchange of blood and CSF between cranial and spinal compartments over the cardiac cycle changes the geometry and dielectric properties of the brain. The Monro-Kellie doctrine (Monro, 1783, Kellie, 1824) stipulates that since the skull is rigid and contains primarily brain parenchyma, CSF and blood, which are incompressible, the sum of their volumes must remain constant. This means that any change in the volume of one component has to be compensated. Therefore, during the systolic part of the cardiac cycle, when the blood volume in the brain increases, CSF volume must decrease, and vice versa during diastole. This is a dynamic and spatially heterogeneous (i.e., location-dependent) process that results in pulsation (changes in shape and location of brain structures and the overall brain) and associated brain dielectric property variations due to varying amount of tissue perfusion. These dynamic changes affect the head’s electric impedance, such that head impedance measurements are likely to include information content that can be related to brain motion dynamics and associated ICP/CC changes. Computational modeling is a powerful tool to help understand and exploit this relationship, not just to establish a non-invasive ICP/CC surrogate, but to advance the knowledge about dynamic ICP and its pathophysiological basis. This demands simulation of an electrostatic problem in which the geometry is dynamic and the dielectric properties vary simultaneously. The quantity-of-interest is the small impedance variation, which must be computed at sufficient temporal resolution over the interval of interest (primarily the cardiac cycle). The natural, direct, and simple solution is to apply suitable boundary conditions to (at least) two surface electrodes and solve the Laplace equation for all time steps, while adapting the geometry and dielectric properties:

(1) $$\nabla \cdot (\overset{\sim }{∊}\nabla \phi (r,t))=0,$$

where $\overset{\sim }{∊}$ and ϕ are the complex permittivity and the scalar electric potential, respectively. This is a quasi-static approximation of Maxwell’s equations which neglects the displacement current. It is suitable in the frequency range of interest, i.e., around 1 MHz (Andreas Spiegelberg et al., 2022), because the head dimensions are much smaller than the EM wavelength (multiple meters). For this study, Dirichlet boundary conditions (constant voltage) were used on the electrodes and insulating Neumann boundary conditions at the remaining surfaces. The base admittance is the reverse of the impedance and obtained as:

(2) $$Y=\frac{1}{Z}=\frac{i\omega Q}{V_{0}},$$

where $Q=\int_{\Omega }\overset{\sim }{∊}E\cdot \mathrm{dS}$ is the total electric charge (E=-∇ϕ: electric field; $V_{0}$: applied voltage; ω: angular frequency; Ω: closed surface encompassing one of the electrodes). The admittance change relative to the base admittance is then computed as:

(3) $$\mathrm{dY}=Y^{*}-Y=i\omega \frac{Q^{*}-Q}{V_{0}}=i\omega \frac{\mathrm{dQ}}{V_{0}},$$

where $Y^{*}$ is the structure admittance at any time point during the cardiac cycle. In this paper, the superscript ^∗^ denotes values after applying geometric or dielectric changes, whereas its absence denotes the initial configuration.

This natural approach suffers from two primary shortcomings: first, the computational cost is high because the Laplace equation must be repeatedly solved for the considered dynamic configuration. Second, the tiny perturbations in the problem specifications lead to similar values for $Y^{*}$ and *Y*, such that any inaccuracy, e.g., related to the simulation, will dominate. Inaccuracies can be reduced by refining the discretization and strengthening the convergence solver criterion, which in turn incurs dramatic increases in computational cost.

**Fig. 1:**
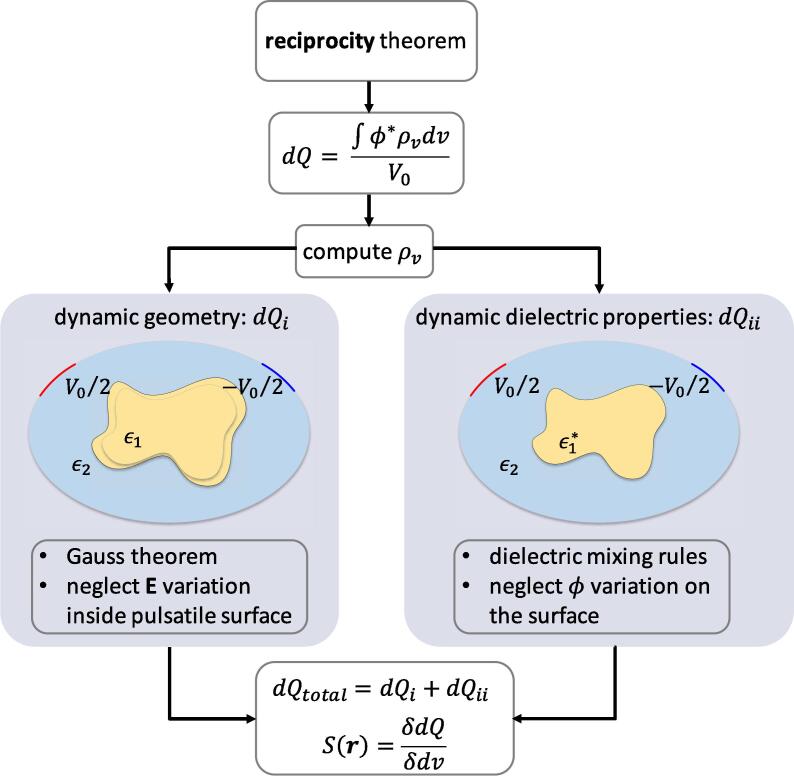
Developed computational pipeline based on the reciprocity theorem for dynamic EM problems. A closed-form equation for determining effective charge layer distributions is derived and used to compute the associated impedance changes (via the electric charge variation *dQ*) as well as sensitivity maps (S(r), functional derivative of *dQ* to local brain pulsation). A sensitivity map provides insight into the spatial distribution of pulsation-contributions to the measurable impedance signal.

Hence, we developed, based on the reciprocity theorem (Plonsey, 1963), a novel approach for computing the quantities-of-interest. Fig. 1 shows an overview of the approach. First, using the reciprocity theorem, a closed-form equation for the dynamic charge change is derived. An effective bi-layer charge distribution is determined, on the basis of which the impact of moving material boundaries - while maintaining dielectric properties fixed - is quantified (case (i)), along with that of changing these properties while maintaining the geometry (case (ii)). The combined, general case is referred to as case (iii).

#### 2.1.1 Reciprocity theorem application

Let $d\phi =\phi^{*}-\phi$ and $d\overset{\sim }{∊}={\overset{\sim }{∊}}^{*}-\overset{\sim }{∊}$ be the perturbations of the scalar electric potential and of the permittivity. The quasi-static Laplace equation for $\phi^{*}$ implies that $\nabla \cdot ({\overset{\sim }{∊}}^{*}\nabla \phi^{*})=0$, which yields:

(4) $$\nabla \cdot ({\overset{\sim }{∊}}^{*}\nabla d\phi )=-\nabla \cdot ({\overset{\sim }{∊}}^{*}\nabla \phi )=-\nabla \cdot (\overset{\sim }{∊}\nabla \phi )-\nabla \cdot (d\overset{\sim }{∊}\nabla \phi )\text{.}$$

$\nabla \cdot (\overset{\sim }{∊}\nabla \phi )$ is zero everywhere, and $\nabla \cdot (d\overset{\sim }{∊}\nabla \phi )$ is only nonzero on the original and the new location of the shifting interfaces. Eq. (4) suggests that instead of computing dϕ as the difference of ϕ and $\phi^{*}$, one can compute dϕ and the corresponding *dQ* directly by solving a corresponding Poisson equation.

While this reduces the accuracy issue, it still demands solving a Poisson problem for each relevant time-point as $d\overset{\sim }{∊}$ changes. To overcome this challenge, the reciprocity theorem (Plonsey, 1963) is applied, which states that: in a volume *V* bounded by surface *S* and having complex permittivity $\overset{\sim }{∊}$, if volume charge densities $\rho_{v1}$ and $\rho_{v2}$ give rise to scalar potentials $\phi_{1}$ and $\phi_{2}$, such that $\nabla \cdot (\overset{\sim }{∊}\nabla \phi_{1})=-\rho_{v1}$ and $\nabla \cdot (\overset{\sim }{∊}\nabla \phi_{2})=-\rho_{v2}$, the following vector identity must be satisfied:

(5) $$\int_{S}\phi_{1}(\overset{\sim }{∊}\nabla \phi_{2})\cdot \mathrm{dS}-\int_{S}\phi_{2}(\overset{\sim }{∊}\nabla \phi_{1})\cdot \mathrm{dS}=\int_{V}\phi_{1}\left(\nabla \cdot (\overset{\sim }{∊}\nabla \phi_{2})\right)\mathrm{dv}-\int_{V}\phi_{2}\left(\nabla \cdot (\overset{\sim }{∊}\nabla \phi_{1})\right)\mathrm{dv}\text{.}$$

By assigning $\phi_{1}=\phi^{*},\phi_{2}=d\phi$, and using Eq. (4), one can write:

(6) $$\int_{S}\phi^{*}({\overset{\sim }{∊}}^{*}\nabla d\phi )\cdot \mathrm{dS}-\int_{S}d\phi ({\overset{\sim }{∊}}^{*}\nabla \phi^{*})\cdot \mathrm{dS}=-\int_{V}\phi^{*}\rho_{v}\mathrm{dv}-0$$

where $\rho_{v}=-\nabla \cdot ({\overset{\sim }{∊}}^{*}\nabla \phi )$, considering that $\nabla \cdot ({\overset{\sim }{∊}}^{*}\nabla \phi^{*})=0$.

As the electric permittivity of the background medium (air) is very small compared to the permittivities of the various head tissues, it can be approximated as zero. Then, $\int_{S}d\phi ({\overset{\sim }{∊}}^{*}\nabla \phi^{*})\cdot \mathrm{dS}=0$because dϕ vanishes on the electrodes (identical boundary conditions for ϕ and $\phi^{*}$) and elsewhere ${\overset{\sim }{∊}}^{*}\nabla \phi^{*}=0$. Additionally, $\int_{S}\phi^{*}({\overset{\sim }{∊}}^{*}\nabla d\phi )\cdot \mathrm{dS}=\int_{S_{E1}}V_{E1}\cdot q\cdot \mathrm{dS}+\int_{S_{E2}}V_{E2}\cdot q\cdot \mathrm{dS}+\int_{S_{\mathrm{Neumann}}}\phi^{*}\cdot 0\cdot \mathrm{dS}=-V_{0}\mathrm{dQ}$, where E1 and E2 denote the electrode regions where Dirichlet boundary conditions are applied, $q={\overset{\sim }{∊}}^{*}\nabla d\phi$ is the charge density, and $S_{\mathrm{Neumann}}$ stands for the boundary surfaces where an isolating zero-flux Neumann boundary conditions is applied. As a result,

(7) $$\mathrm{dQ}=\frac{\int \phi^{*}\rho_{v}\mathrm{dv}}{V_{0}}\text{.}$$

**Fig. 2:**
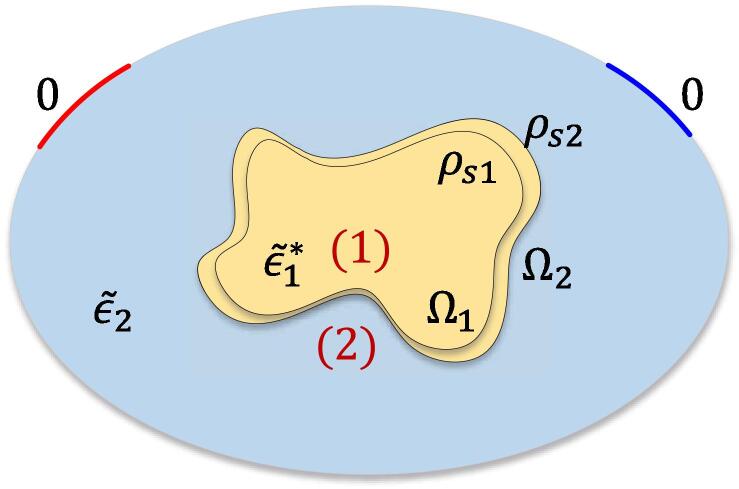
Sketch illustrating a dynamic EM problem with a shifting interface. The impedance computation due to the dynamic geometry changes is mathematically recast (using the reciprocity theorem) into the task of determining equivalent dipolar charge-density distributions residing at the shifting interface. Numerical methods are then developed to efficiently estimate those distributions.

Without loss of generality, we assume that there is only one shifting interface in the geometry, which moves from region 1 towards region 2 (Fig. 2). Then, $\rho_{v}$ can be written as

(8) $$\rho_{v}=\rho_{s1}\delta \left(r-r_{1}\right)+\rho_{s2}\delta \left(r-r_{2}\right)$$

where $\rho_{s1}$ and $\rho_{s2}$ are surface charge densities, and $r_{1}$ and $r_{2}$ are position vectors for points located on the original and new location of the interface, respectively. In other words, the Poisson equation source term $-\nabla \cdot ({\overset{\sim }{∊}}^{*}\nabla \phi )$ effectively behaves like a charge bi-layer. Using Gauss’ theorem and the conservation of current density on the interface, $\rho_{s1}$ and $\rho_{s2}$ are obtained as:

(9) $$\begin{array}{cc} \rho_{s1} & =\left(\overset{\sim }{{∊}_{2}}\frac{{\overset{\sim }{{∊}_{1}}}^{*}}{\overset{\sim }{{∊}_{1}}}-{\overset{\sim }{{∊}_{1}}}^{*}\right)\frac{\partial \phi }{\partial n}{|}_{\Omega_{1}^{+}},\\ \rho_{s2} & =-\left(\overset{\sim }{{∊}_{2}}-{\overset{\sim }{{∊}_{1}}}^{*}\right)\frac{\partial \phi }{\partial n}{|}_{\Omega_{2}}, \end{array}$$

where *n* is the normal vector to the interface, + denotes the side of interface within the second region, and $\Omega_{1}$ and $\Omega_{2}$ denote the original and the new location of the interface, respectively. Substituting Eq. (9) in Eq. (7) yields

(10) $$\mathrm{dQ}=\frac{\int_{\Omega_{1}}\phi^{*}(r_{1})\rho_{s1}\mathrm{dS}+\int_{\Omega_{2}}\phi^{*}(r_{2})\rho_{s2}\mathrm{dS}}{V_{0}}\text{.}$$

#### 2.1.2 Solution algorithm for case (i)

Case (i) assumes that the dielectric properties remain constant while the interface moves. Thanks to the reciprocity theorem, *dQ* is computed directly through a more stable numerical procedure given in Eq. (10). However, the dependence on $\phi^{*}$ still demands repeated solving of the quasi-static Laplace equation for all relevant configurations and time-points. Furthermore, extracting ϕ and $\frac{\partial \phi }{\partial n}$ on two interfaces with tiny separation can also result in a large numerical error. Hence, a first approximation is introduced. It relies on small interface shifts: Eq. (9), Eq. (10), and the Gauss’ theorem lead to

(11) $$\begin{array}{cc} V_{0}\mathrm{dQ} & =\int_{\Omega_{1}}(\overset{\sim }{{∊}_{2}}-{\overset{\sim }{∊}}_{1})\phi^{*}(r_{1})\frac{\partial \phi }{\partial n}{|}_{\Omega_{1}^{+}}\mathrm{dS}-\int_{\Omega_{2}}(\overset{\sim }{{∊}_{2}}-\overset{\sim }{{∊}_{1}})\phi^{*}(r_{2})\frac{\partial \phi }{\partial n}{|}_{\Omega_{2}}\mathrm{dS}\\ & =-\int_{\Omega_{1},\Omega_{2}}(\overset{\sim }{{∊}_{2}}-\overset{\sim }{{∊}_{1}})\phi^{*}\nabla \phi \cdot dS=-\int_{v}(\overset{\sim }{{∊}_{2}}-\overset{\sim }{{∊}_{1}})\nabla \cdot (\phi^{*}\nabla \phi )\mathrm{dv}\\ & =-\int_{v}(\overset{\sim }{{∊}_{2}}-\overset{\sim }{{∊}_{1}})E^{*}\cdot E\mathrm{dv}=-\int_{\Omega_{1}}\int_{r_{1}}^{r_{2}}(\overset{\sim }{{∊}_{2}}-\overset{\sim }{{∊}_{1}})E^{*}\cdot \mathrm{Edr}\cdot \mathrm{dS}\\ & \approx -\int_{\Omega_{1}}(\overset{\sim }{{∊}_{2}}-\overset{\sim }{{∊}_{1}})E_{+}^{*}\cdot E_{+}\left(\int_{r_{1}}^{r_{2}}\mathrm{dr}\right)\cdot \mathrm{dS}=-\int_{\Omega_{1}}(\overset{\sim }{{∊}_{2}}-\overset{\sim }{{∊}_{1}})E_{+}^{*}\cdot E_{+}\left(r_{2}-r_{1}\right)\cdot \mathrm{dS}, \end{array}$$

where $\overset{\sim }{{∊}_{1}}$ and $\overset{\sim }{{∊}_{2}}$ are the complex permittivity of region 1 and region 2, respectively. In the last step, we neglect the E and $E^{*}$ variations between the original and new location of the interface.

On the interface $\Omega_{1},\;{\mathrm{dE}}_{t}^{+}={\mathrm{dE}}_{t}^{-}$ and according to Eq. (4), ${\mathrm{dE}}_{n}^{+}-{\mathrm{dE}}_{n}^{-}=-\frac{\rho_{s1}}{\overset{\sim }{{∊}_{1}}}$, with $E_{t}$ and $E_{n}$ being the tangential and normal components of electric field, respectively. Therefore, by assuming ${\mathrm{dE}}^{-}≅0$ (due to the fact that the difference between E and $E^{*}$ in region 1 is negligible), one can write:

(12) dE+=-ρs1∊1~n^

and

(13) E+∗=E++dE+=E+-ρs1∊1~n^

By substituting Eq. (13) in Eq. (11), we obtain:

(14) $$\begin{array}{cc} V_{0}\mathrm{dQ} & =-\int_{\Omega_{1}}\left((\overset{∼}{{∊}_{2}}-\overset{∼}{{∊}_{1}})|E_{+}{|}^{2}+\frac{\rho_{s1}^{2}}{\overset{∼}{{∊}_{1}}}\right)\left(r_{2}-r_{1}\right)\cdot \mathrm{dS}\\ & =-\int_{\Omega_{1}}\left((\overset{∼}{{∊}_{2}}-\overset{∼}{{∊}_{1}})\left(|E_{n}^{+}{|}^{2}+|E_{t}{|}^{2}\right)+\frac{{\left(\overset{∼}{{∊}_{2}}-\overset{∼}{{∊}_{1}}\right)}^{2}}{\overset{∼}{{∊}_{1}}}|E_{n}^{+}{|}^{2}\right)\left(r_{2}-r_{1}\right)\cdot \mathrm{dS}\\ & =-\int_{\Omega_{1}}\left(\left(\frac{{\overset{∼}{{∊}_{2}}}^{2}}{\overset{∼}{{∊}_{1}}}-\overset{∼}{{∊}_{2}}\right)|E_{n}^{+}{|}^{2}+\left(\overset{∼}{{∊}_{2}}-\overset{∼}{{∊}_{1}}\right)|E_{t}{|}^{2}\right)\left(r_{2}-r_{1}\right)\cdot \mathrm{dS}\text{.} \end{array}$$

Note that it is easily shown that Eq. (14) holds independently of the movement direction of the interface, i.e., whether it moves toward region one or two.

#### 2.1.3 Solution algorithm for case (ii)

Case (ii) assumed that the geometry is constant while the dielectric properties change as a result of the varying amount of brain tissue perfusion over the cardiac cycle. Based on the Monro-Kellie doctrine, the change in intracranial blood volume is thought to exactly compensate that of intracranial CSF. The corresponding effective source in the Poisson equation is then reduced to a single charge density layer at the interface, i.e., $\rho_{v}=\rho_{s}\delta \left(r-r_{1}\right)$. Under these circumstances, *dQ* is obtained as follows:

(15) $$\begin{array}{cc} \mathrm{dQ} & =\frac{\int_{\Omega_{1}}\phi^{*}(r_{1})\rho_{s1}\mathrm{dS}}{V_{0}}=\frac{\int_{\Omega_{1}}({\overset{\sim }{∊}}_{1}^{*}-{\overset{\sim }{∊}}_{1})\phi^{*}(r_{1})\frac{\partial \phi }{\partial n}{|}_{\Omega_{1}^{-}}\mathrm{dS}}{V_{0}}=({\overset{\sim }{∊}}_{1}^{*}-{\overset{\sim }{∊}}_{1})\frac{\int_{\Omega_{1}}\phi^{*}(r_{1})\frac{\partial \phi }{\partial n}{|}_{\Omega_{1}^{-}}\mathrm{dS}}{V_{0}}\\ & \approx d{\overset{\sim }{∊}}_{1}\frac{\int_{\Omega_{1}}\phi (r_{1})\frac{\partial \phi }{\partial n}{|}_{\Omega_{1}^{-}}\mathrm{dS}}{V_{0}}, \end{array}$$

where the approximation $\phi^{*}(r_{1})\approx \phi (r_{1})$ is used in the last step, which is valid for small dielectric property changes.

Estimating the $d\overset{\sim }{∊}$ resulting from brain perfusion changes requires a suitable material model. Dielectric mixing rules are algebraic formulas that derive the permittivity of a mixture as a function of its components’ permittivities and their fractional volumes. They are mostly applicable in the long-wavelength regime, which is also a condition for the quasi-static approximation to be valid and is justified at the operation frequency of ∼1 MHz, relevant to the application-of-interest.

Several mixing rules have been proposed, including the Clausius–Mossotti formula (Sihvola, 2000, Van Rysselberghe, 2002, Peter Atkins et al., 2010), the Rayleigh formula (Sihvola, 2000), the Maxwell–Garnett formula (Sihvola, 2000), the Bruggeman formula (Bruggeman, 1935, Karkkainen et al., 2000), and the Lichtenecker formula (Karkkainen et al., 2000, von Lichtenecker, 1931). For the small perfusion-change-related variations of interest (fractional change <0.1% (Gehlen et al., 2017)), the differences in $d\overset{\sim }{∊}$ obtained using different mixing rules were found to be small (the variability in $d\overset{\sim }{∊}$ among the nine considered models was below 17%). Thus, the Lichtenecker formula was used, as it is the most common model for biological tissues in literature. It implies that:

(16) $${\overset{\sim }{∊}}_{\mathrm{eff}}={\overset{\sim }{∊}}_{\mathrm{tissue}}^{1-\frac{{\mathrm{dv}}_{\mathrm{total}}}{V_{\mathrm{tissue}}}}{\overset{\sim }{∊}}_{\mathrm{blood}}^{\frac{{\mathrm{dv}}_{\mathrm{total}}}{V_{\mathrm{tissue}}}}$$

where ${\mathrm{dv}}_{\mathrm{total}},V_{\mathrm{tissue}},{\overset{\sim }{∊}}_{\mathrm{tissue}}$, and ${\overset{\sim }{∊}}_{\mathrm{blood}}$ are the blood volume variation, the total tissue volume, its complex permittivity, and that of blood, respectively. As a result:

(17) $$d\overset{\sim }{{∊}_{1}}={\overset{\sim }{∊}}_{\mathrm{eff}}-{\overset{\sim }{∊}}_{\mathrm{tissue}}={\overset{\sim }{∊}}_{\mathrm{tissue}}\left({\left(\frac{{\overset{\sim }{∊}}_{\mathrm{blood}}}{{\overset{\sim }{∊}}_{\mathrm{tissue}}}\right)}^{\frac{{\mathrm{dv}}_{\mathrm{total}}}{V_{\mathrm{tissue}}}}-1\right)\approx {\overset{\sim }{∊}}_{\mathrm{tissue}}\left(\frac{{\mathrm{dv}}_{\mathrm{total}}}{V_{\mathrm{tissue}}}\ln\left(\frac{{\overset{\sim }{∊}}_{\mathrm{blood}}}{{\overset{\sim }{∊}}_{\mathrm{tissue}}}\right)\right),$$

which leads to:

(18) $$V_{0}\mathrm{dQ}={\overset{\sim }{∊}}_{\mathrm{tissue}}\left(\frac{{\mathrm{dv}}_{\mathrm{total}}}{V_{\mathrm{tissue}}}\ln\left(\frac{{\overset{\sim }{∊}}_{\mathrm{blood}}}{{\overset{\sim }{∊}}_{\mathrm{tissue}}}\right)\right)\int_{\Omega_{1}}\phi (r_{1})\frac{\partial \phi }{\partial n}{|}_{\Omega_{1}^{-}}\mathrm{dS}\text{.}$$

#### 2.1.4 Solution algorithm for case (iii)

When the geometry and the dielectric properties vary simultaneously, the combined impact of small variations can be obtained using:

(19) $$\mathrm{dQ}(v,\overset{\sim }{∊})=\frac{\partial Q(v,\overset{\sim }{∊})}{\partial \mathrm{dv}}\mathrm{dv}+\frac{\partial Q(v,\overset{\sim }{∊})}{\partial \overset{\sim }{∊}}d\overset{\sim }{∊}\text{.}$$

Thus, *dQ* can be computed as follows:

(20) $$V_{0}\mathrm{dQ}=-\int_{\Omega_{1}}\left(\left(\frac{{\overset{\sim }{{∊}_{2}}}^{2}}{\overset{\sim }{{∊}_{1}}}-\overset{\sim }{{∊}_{2}}\right)|E_{n}^{+}{|}^{2}+\left(\overset{\sim }{{∊}_{2}}-\overset{\sim }{{∊}_{1}}\right)|E_{t}{|}^{2}\right)\left(r_{2}-r_{1}\right)\cdot \mathrm{dS}+d\overset{\sim }{{∊}_{1}}\int_{\Omega_{1}}\phi (r_{1})\frac{\partial \phi }{\partial n}{|}_{\Omega_{1}^{-}}\mathrm{dS}\text{.}$$

Eq. (20) shows that it is possible to compute *dQ*, for small changes in geometry and dielectric properties using a single simulation per electrode configuration, and only necessitating knowledge about the electric field on the original interface location, resulting in a numerically robust and computationally efficient procedure for computing *dY*. For the case of the human head, substituting Eq. (17) in Eq. (20) yields:

(21) $$\begin{array}{cc} V_{0}\mathrm{dQ} & =-\int_{\Omega_{1}}\left(\left(\frac{{\overset{\sim }{{∊}_{2}}}^{2}}{\overset{\sim }{{∊}_{1}}}-\overset{\sim }{{∊}_{2}}\right)|E_{n}^{+}{|}^{2}+\left(\overset{\sim }{{∊}_{2}}-\overset{\sim }{{∊}_{1}}\right)|E_{t}{|}^{2}\right)\left(r_{2}-r_{1}\right)\cdot \mathrm{dS}\\ & +{\overset{\sim }{∊}}_{\mathrm{tissue}}\left(\frac{{\mathrm{dv}}_{\mathrm{total}}}{V_{\mathrm{tissue}}}\ln\left(\frac{{\overset{\sim }{∊}}_{\mathrm{blood}}}{{\overset{\sim }{∊}}_{\mathrm{tissue}}}\right)\right)\int_{\Omega_{1}}\phi (r_{1})\frac{\partial \phi }{\partial n}{|}_{\Omega_{1}^{-}}\mathrm{dS}\text{.} \end{array}$$

#### 2.1.5 Sensitivity

The contribution of a local displacement to the admittance change is quantified by the sensitivity map S(r), which is defined as a functional derivative (Parr et al., 1994) $\frac{\partial \mathrm{dQ}}{\partial \mathrm{dv}}$ and

(22) $$\mathrm{dQ}=\int \frac{\delta \mathrm{dQ}}{\delta \mathrm{dv}}\mathrm{dv}=\int \frac{\delta \mathrm{dQ}}{\delta \mathrm{dv}}\mathrm{dr}\cdot \mathrm{dS}\text{.}$$

Sensitivity maps can be used to optimize the measurement electrode configuration such that the information content about CC is maximized.

Combing Eq. (20) and Eq. (22) and substituting Eq. (17) yields:

(23) $$\begin{array}{cc} S(r) & =-\left(\left(\frac{{\overset{\sim }{{∊}_{2}}}^{2}}{\overset{\sim }{{∊}_{1}}}-\overset{\sim }{{∊}_{2}}\right)|E_{n}^{+}{|}^{2}+\left(\overset{\sim }{{∊}_{2}}-\overset{\sim }{{∊}_{1}}\right)|E_{t}{|}^{2}\right)\\ & +{\overset{\sim }{∊}}_{\mathrm{tissue}}\left(\frac{1}{V_{\mathrm{tissue}}}\ln\left(\frac{{\overset{\sim }{∊}}_{\mathrm{blood}}}{{\overset{\sim }{∊}}_{\mathrm{tissue}}}\right)\right)\int_{\Omega_{1}}\phi (r_{1})\frac{\partial \phi }{\partial n}{|}_{\Omega_{1}^{-}}\mathrm{dS}\text{.} \end{array}$$

### 2.2 Benchmarks

**Fig. 3:**
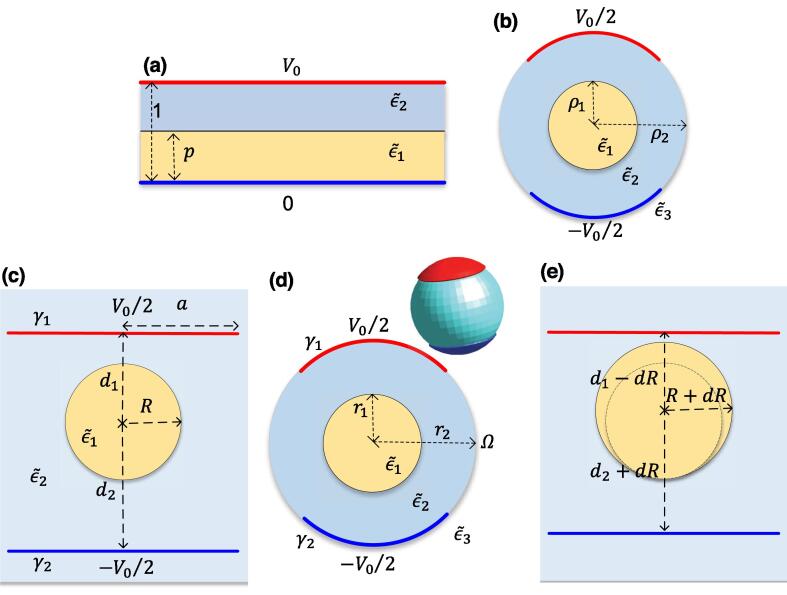
Geometries of the (a) 1D, (b) 2D symmetric, (c) 2D asymmetric, and (d) 3D benchmarks; (e) geometry changes in the 2D asymmetric benchmark.

To verify the developed formula and investigate the correctness of proposed approximation, four analytically or semi-analytically solvable benchmarks were established (Fig. 3).

#### 2.2.1 1D benchmark

For the first benchmark, we propose a simple 1D model consisting of two dielectric slabs with different permittivities sandwiched between two electrodes as shown in Fig. 3a. The derivation of the analytical solution of Eq. (1) in this geometry and the associated electric charge density (*Q*) are discussed in Supplement 1.1.

Fig. S1 shows results for $\overset{\sim }{{∊}_{r1}}=2,\overset{\sim }{{∊}_{r2}}=10$, and p=0.5, along with the relative error $\left|\frac{{\mathrm{dQ}}_{\mathrm{direct}}-{\mathrm{dQ}}_{\mathrm{formula}}}{{\mathrm{dQ}}_{\mathrm{direct}}}\right|$, where ${\mathrm{dQ}}_{\mathrm{direct}}=Q-Q^{*},Q$ and $Q^{*}$ are the electric charge densities before and after interface motion and/or $\overset{\sim }{{∊}_{1}}$ changes, and ${\mathrm{dQ}}_{\mathrm{formula}}$ is the estimation of *dQ* obtained using Eq. (21). The relative error for slab thickness and permittivity changes of up to 10% always remained below 7%.

#### 2.2.2 2D symmetric benchmark

Fig. 3b illustrates the 2D symmetric benchmark. Using the general solution of the Laplace equation in cylindrical coordinates and enforcing the boundary conditions on $\rho =\rho_{1}$ and $\rho =\rho_{2}$, namely electric potential continuity and conservation of the normal component of current, the electric potential can be determined semi-analytically (see Supplement 1.2).

First, the semi-analytical solution was verified against simulation results obtained using the finite-difference method solver of Sim4Life V6.3 (ZMT Zurich MedTech AG, Switzerland; see Supplement 1.2). Subsequently, $\mathrm{dQ}=Q-Q^{*}$ with $Q^{*}$ calculated after the interface moves and $\overset{\sim }{{∊}_{1}}$ changes was compared with the *dQ* estimation from Eq. (21) (see Fig. S3). The relative error for radius and permittivity changes of up to 10% always remained below 2.5%.

#### 2.2.3 2D asymmetric benchmark

As the two previous benchmarks have a high degree of symmetry that might mask false assumptions in the derivation of the reciprocity theorem approach (e.g., because the displacement vector is always normal to the interface), a benchmark lacking such symmetries was derived (see Fig. 3c). Image theory was applied to solve this benchmark analytically (see Supplement 1.3 for details of the solution).

The analytical solution was again verified against simulations (see Supplement 1.3), before using it to validate the reciprocity-theorem-based approach for cases where the interface movement direction is not aligned with the normal vector to the interface (Fig. 3e). The results are shown in Fig. S6. The relative error for radius and permittivity changes of up to 10% always remained below 5%. In addition, the 2D asymmetric benchmark was used to validate the applicability of the developed method in the case where interface motion results in direct contact with another tissue (dura). CSF and brain properties were assigned and the radius was varied such that in one case contact with the boundary was established, and in another case not. The relative difference between *dQ* from the semi-analytical solution and the developed method (Eq.(21)) is only 2.3% for a radius variation of 5%.

#### 2.2.4 3D benchmark

Fig.3d shows the geometry of the 3D benchmark. Using the general solution of Laplace equation in spherical coordinates and enforcing the boundary conditions at $r=r_{1}$ and $r=r_{2}$, we obtained an analytical expression for the electric potential (see Supplement 1.4), which was verified against simulation results and used to validate the reciprocity-theorem-based approach in full 3D (see Fig. S8). The relative error never exceeded 6%.

### 2.3 Detailed and realistic model

To study the impedance changes related to geometry and dielectric property variations in realistic scenarios, simulations were performed using Sim4Life’s unstructured electro-quasistatic (EQS) solver, which employs the finite element method (FEM). These simulations involved a detailed anatomical head model, along with 4D deformation data obtained from healthy volunteers. Based on Eq. (21) and using the sensitivity map Eq. (23), *dQ* can then be computed if information about brain surface pulsation is available. Primarily because of poor data quality at the brain surface, a number of preprocessing steps are required to extract this information from the 4D deformation data.

#### 2.3.1 EM simulations with the MIDA model

**Fig. 4:**
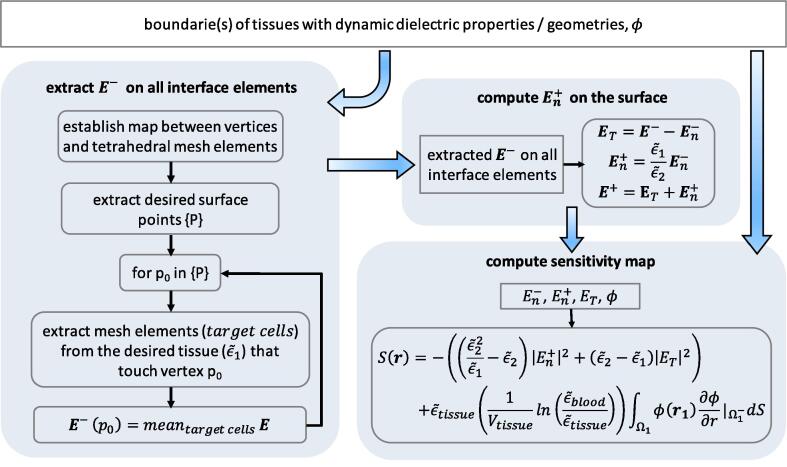
Simulation post-processing pipeline used for EM simulations with the MIDA model.

EM simulations were performed using the MIDA model (Iacono et al., 2015). The MIDA model is a high-resolution, detailed anatomical model of the human head and neck, based on multimodal image data from a healthy volunteer, which distinguishes 115 different tissues. In a first step, a tetrahedral mesh of the MIDA with 2.2 million elements was created using Sim4Life, along with two meshes with 4 and 10 million elements for the grid convergence analysis. The dielectric tissue properties were assigned based on the IT’IS database (Hasgall et al., 2018). To solve Eq. (1), the unstructured EQS solver was used. Dirichlet boundary conditions were applied to the rectangular $16\times 16\;\;{\mathrm{mm}}^{2}$ electrodes. The working frequency was chosen to be 1 MHz based on the recently developed device by Andreas Spiegelberg et al. (2022). Then, Eq. (23) was used to compute the sensitivity maps. The post-processing pipeline shown in Fig. 4 was implemented to overcome associated numerical challenges.

#### 2.3.2 Deformation data

*Acquisition.* Deformation data is typically acquired using different MRI techniques, i.e., displacement encoding with stimulated echoes (DENSE) (Soellinger et al., 2009, Adams et al., 2020) and phase-contrast MRI (PC-MRI) (Enzmann et al., 1992, Dan Greitz et al., 1992). For this study, existing DENSE data from eight healthy young subjects of European descent acquired using a 7T MR scanner was used (Adams et al., 2020). The whole cardiac cycle was covered in 20 snapshots, for two opposite gradient polarities. Background errors were removed by subtracting the data from those two polarities.

**Fig. 5:**
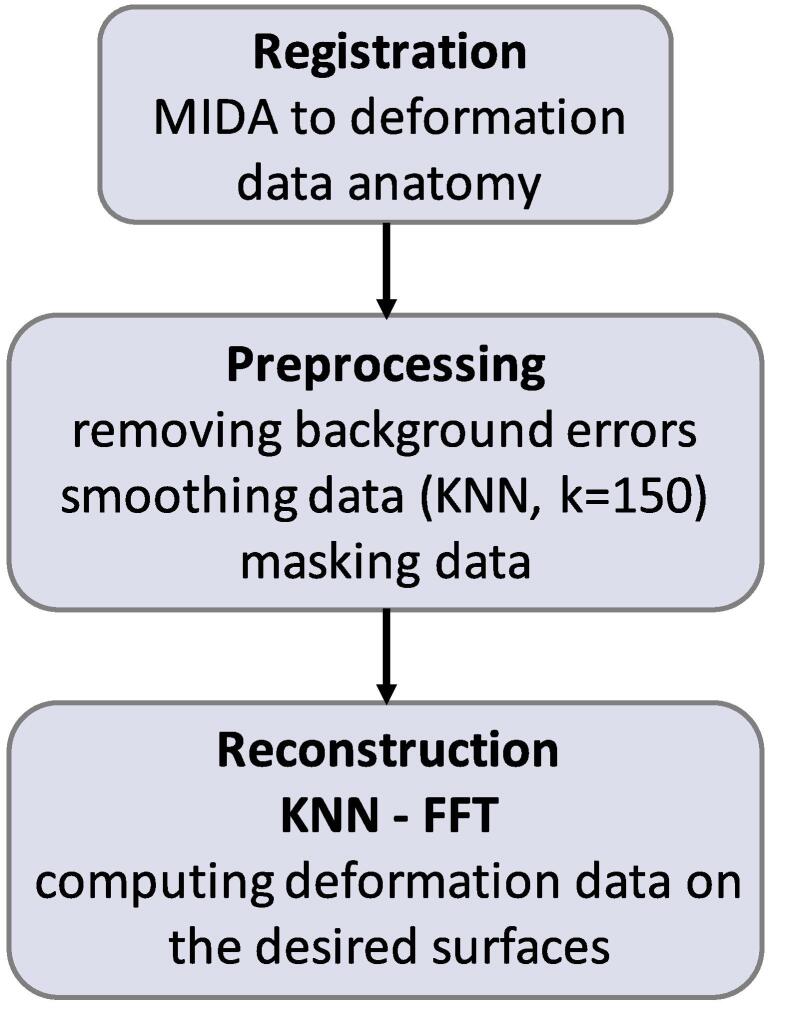
Schematic description of the pipeline used to process the imaging-based deformation data in preparation for their combination with the computationally determined sensitivity maps.

*Processing.*Fig. 5 shows the developed pipeline for deformation data analysis. As the anatomical model was generated for a head anatomy different from that of the DENSE imaged subjects, (manual) *registration* was performed by first shifting and rotating the MIDA model to align its brain regions with the deformation data (primarily relying on brain surface, ventricles, and eye landmarks). Subsequently, automatic optimization of the MIDA model scaling along the x, y, and z directions was performed, minimizing the absolute value of the relative difference between the brain volume of these two different anatomies. Finally, another fine-correction of the shift was performed when required.

**Fig. 6:**
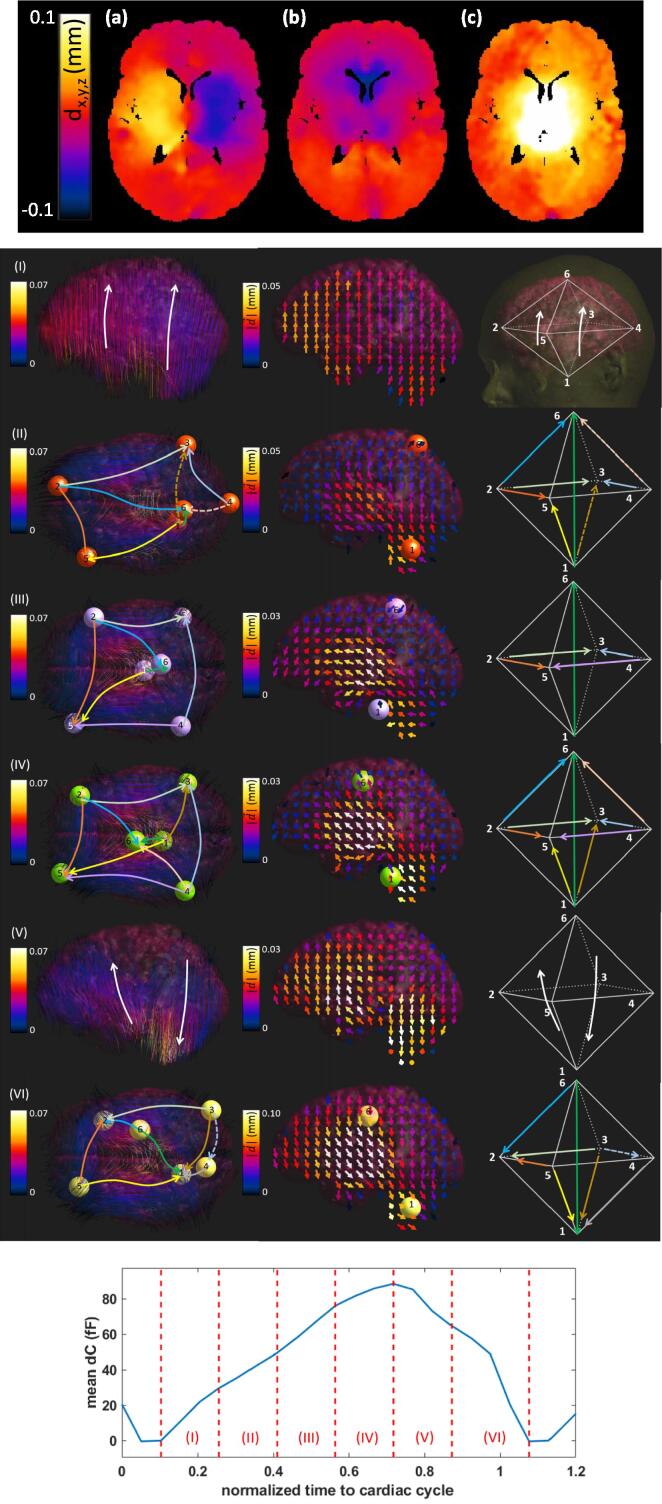
(Top) Illustrative KNN-smoothed deformation data from one subject at one time point during the cardiac cycle; (a) right-left, (b) anterior-posterior, and (c) cranial-caudal component. (Middle) Illustration of the brain deformation over one cardiac cycle (the coloring denotes the motion magnitude); (left) streamlines and principal motion trajectories, (middle) vector field views, (right) schematic representation of the overall brain deformation pattern; see Section 3 for extended explanation and interpretation and Fig. S9 for an animated 3D visualization of the streamlines of sub-interval (II). (Bottom) Time-intervals of the pulsation period corresponding to the motion visualizations in the Middle.

The deformation data was masked to remove image regions with high associated noise and/or uncertainty which unfortunately includes the CSF and nearby regions of primary interest to this study. The initial mask encompassed gray matter (GM) and white matter (WM) regions based on CSF, GM, and WM probability maps obtained using SPM12 (Wellcome Centre for Human Neuroimaging, University College London; Adams et al., 2020). That mask was eroded (three voxels) to remove regions near CSF. The k-nearest neighbors algorithm (KNN, scikit-learn) (k = 150) was used to smooth the deformation data and reduce noise (see Fig. 6). To extract the deformation fields on the moving interfaces of interest, the masked data was extrapolated. Different extrapolation methods were compared and were found to result in large differences on the outer brain surface (interpolation to ventricular surfaces was robust). After excluding schemes that resulted in overly large deformation predictions (i.e., ≫100μm), further analysis focused on nearest neighbour extrapolation and fast Fourier transform (FFT). Using FFT as a reconstruction method is not trivial as the masked data is unstructured (due to the masking step) and, to our knowledge, there is no available toolbox for unstructured FFT that does not assume zero outside the domain. To address this issue, we developed a computationally and memory efficient unstructured FFT method (Karimi et al., 2022) that includes regularization based on singular value decomposition (SVD) to handle the associated conditioning problem.

*Visualization.* To visualize the overall brain motion, the periodicity interval (cardiac cycle) was divided into six sub-intervals and the brain surface motion fields were computed by subtracting the displacement vectors at the beginning of a sub-interval from those at the end. Their visualization as vector field, as well as the associated streamlines, which can be thought of as the ‘rails’ of the motion during the sub-interval, can be seen in Fig. 6.

### 2.4 Impedance change computation

**Fig. 7:**
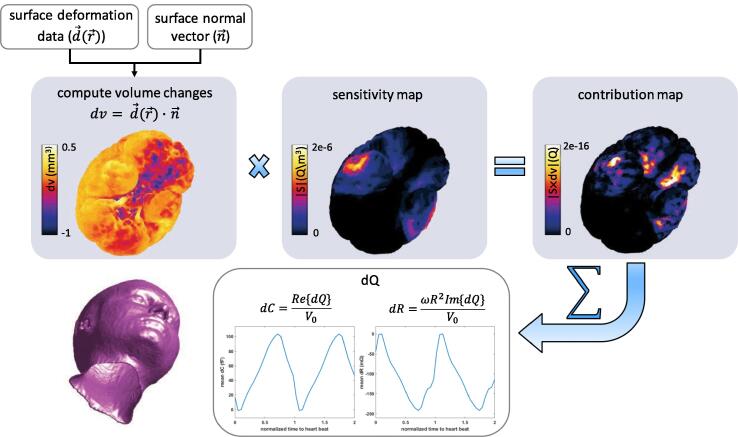
Coupling MIDA-based EM simulations and MRI deformation data to compute *dC* and *dR*. The displacement and, therefore, the signal contribution maps are time dependent and only a snapshot is shown (see Fig. S10 for an animated, transient version).

To estimate the transient variations of the electric charge (*dQ*) and the associated capacitance (*dC*) and resistance (*dR*), the sensitivity map is combined with the surface deformation data (see Section 2.3.1 and Section 2.3.2) using Eq. (21). Fig. 7 illustrates this procedure, which was performed for each subject and electrode configuration.

Inter-subject variability of *dC* and *dR* was further investigated. To compensate for potential shifts in the reference time point (the third MRI snapshot in the cardiac cycle) and separate signal magnitude changes related to aspects such as head size and skull thickness from the signal shape of interest, an offset and scaling factor were determined using least square fitting to the mean curve, which is the average of eight healthy subjects (i.e., $d_{i}^{*}(t)=f_{d,i}\cdot d_{i}(t)+g_{d,i}$, where *d* can stand for *dC* or $\mathrm{dR},\;d^{*}$ for scaled *dC* or *dR*, and *i* for the subject). The standard deviation of the scaling factors $f_{d,i}$ was computed as a measure of the amplitude variability between subjects.

### 2.5 Uncertainty assessment

An uncertainty analysis was performed to investigate the impact of discretization and uncertainty about the underlying dielectric tissue properties on the quantities-of-interest (peak-to-peak variation of *dC* and *dR*). The discretization error was estimated by first performing two successive grid-refinement steps (from 2 to 4, and to 10 million cells). Then, the *dC* and *dR* values at infinitely fine resolution were estimated, by ensuring that the log–log plot of the discretization errors as a function of resolution became linear ($R^{2}\approx 1$). To determine the *dC* and *dR* prediction sensitivity to uncertainties about dielectric tissue properties, the permittivity and conductivity of eleven principal tissues were individually varied (see Table 3) by 20% and sensitivity coefficients were determined ($s_{d,\sigma /∊,t}$ in [dB/dB], where *t* stands for the tissue and *d* for *dC* or *dR*). From these sensitivities, a combined uncertainty was computed assuming log-normal uncertainty distributions with 10% standard deviation for the underlying dielectric tissue properties, according to Taylor et al., 1297, Walter Bich et al., 2006:

(24) $$u_{d}=\sqrt{\underset{t\in \mathrm{tissues}\;}{\sum }\underset{i\in \{\sigma ,∊\}}{\sum }{\left(s_{d,i,t}\cdot u_{i,t}\right)}^{2}}\text{.}$$

### 2.6 Relating head impedance changes and brain pulsation features

Towards establishing a head-impedance-based CC surrogate, the relationship between brain pulsation features and measurable impedance signals (dC(t) and dR(t) for different electrode locations) must be investigated, and the ability of inferring one from the other must be studied. We focused on three important features of deformation data: CSF volume change (${\mathrm{dV}}_{\mathrm{CSF}}$), brain translation ($T_{S}$) and rotation ($R_{S}$), which are determined from the reconstructed surface motion. First, correlations between the transient impedance signal and the transient pulsation features were studied. Where linear correlations are weak, recurring non-linear relationships with hysteretic behavior are observed (‘loops’), motivating the treatment of time-series as point in high-dimensional spaces that can be studied using principal component analysis (PCA; in that way, the components capture the principal dimensions of the variability in the loop shape). The principal components from the concatenated measurement and pulsation vectors of all eight subjects were extracted. Due to the lack of orthogonality of the measurement signal parts, relationships between them and the deformation feature parts were derived using the ridge regression method in Matlab (MATLAB Release 2019b, The MathWorks, Inc., Natick, Massachusetts, United States) and used to ‘predict’ deformation features from the measurable signals. The agreement between these personalized predictions of deformation feature variations and their actual value (as extracted from the subjects raw deformation data) were quantified.

A statistical analysis was performed to investigate whether the measurement signal based predictions are indeed able to account for inter-subject variability and outperform non-personalized approaches. As a Shapiro–Wilk test revealed that the data distribution is not normal, the Wilcoxon signed-rank test was employed (a *p*-value<5% was deemed to be statistically significant).

## 3 Results

**Fig. 8:**
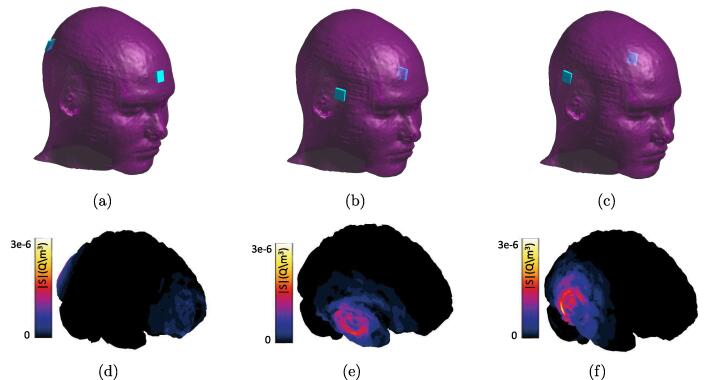
The (a) 1^st^, (b) 2^nd^, and (c) 3^rd^ electrode configuration and the corresponding sensitivity magnitude (|S|) on the cortex ((d)-(f): total sensitivity combining geometry pulsation and dielectric property change contributions – the latter is uniform, as a result of the homogeneous blood distribution assumption, and much smaller than the former).

For three electrode pair configurations on the MIDA Model (see Fig.8), the *head capacitance (C) and resistance (R)* at 1 MHz were determined (Table 1). Table 1 also reports the magnitude of C and R variations resulting from a constant brain surface displacement by 0.1 mm outwards. *Sensitivity maps* according to Eq. (23) were computed on the ventricular and cortical surfaces (see Fig. 8). *Transient capacitance (dC) and resistance (dR) changes* over the cardiac cycle were computed according to the procedure shown in Fig. 7, using experimentally obtained deformation data from eight healthy subjects (reconstructed on the brain surface using the approach from Section 2.3.2).

**Table 1:** Head impedance (capacitance and resistance) and their change (*dC* and *dR*) due to variations in geometry (constant normal displacement by 0.1 mm) and consequently dielectric properties for the three electrode pair configurations.

**pair**	**capacitance (pF)**	**resistance (**Ω**)**	**cortex**		**ventricles**	
			dC (fF)	dR (mΩ)	dC (fF)	dR (mΩ)
**1^st^**	117	793	−490	2544	−14	2
**2^nd^**	182	512	−655	1575	−18	40
**3^rd^**	167	548	−812	2399	−15	43

**Fig. 9:**
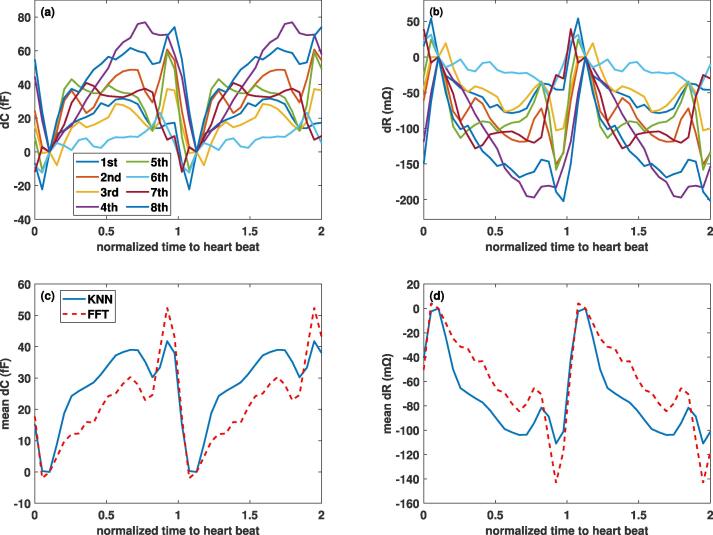
Simulated capacitance (left) and resistance (right) variations associated with the cortical CSF-brain interface, shown over the cardiac cycle for the 2^nd^ electrode configuration. (Top) *dC* and *dR* from data of all eight subjects, using the KNN extrapolation method; (Bottom) comparison between predictions obtained using KNN and FFT reconstruction for the average of all subjects.

**Fig. 10:**
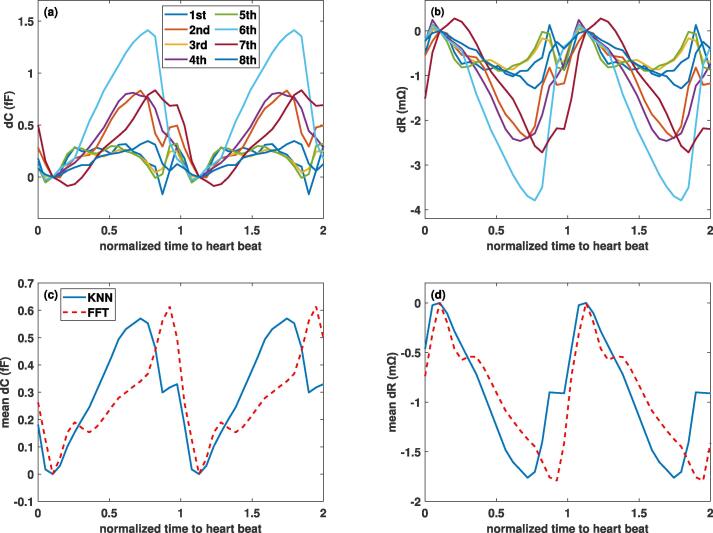
Simulated capacitance (left) and resistance (right) variations associated with the ventricular CSF-brain interface, shown over the cardiac cycle for the 2^nd^ electrode configuration. (Top) *dC* and *dR* from data of all eight subjects, using the KNN extrapolation method; (Bottom) comparison between predictions obtained using KNN and FFT reconstruction for the average of all subjects.

**Fig. 11:**
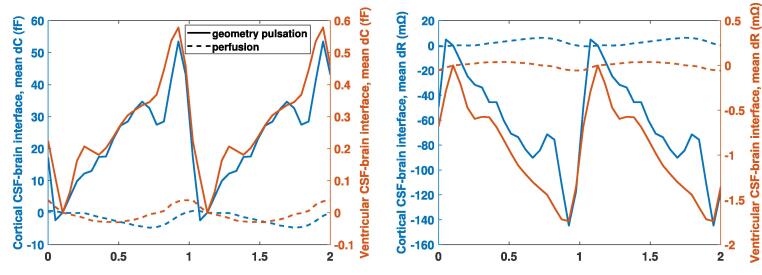
Comparison of the geometry-pulsation- and the blood-perfusion-contributions to the capacitance (left) and resistance (right) variations (average over all subjects, two cardiac cycles, 2^nd^ electrode configuration, FFT-reconstructed deformation data). The contributions from the cortical and ventricular CSF-brain interfaces are distinguished. Note the difference in scaling for ventricular vs. cortical CSF-brain interfaces.

**Fig. 12:**
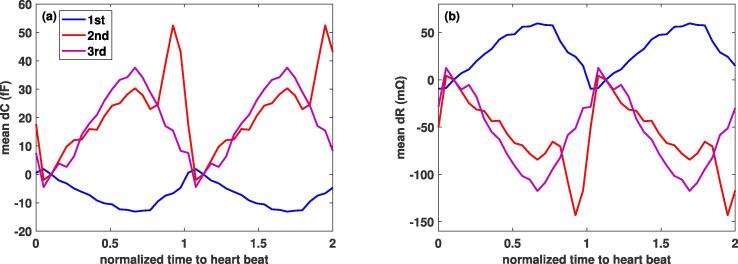
Comparison across the three electrode configurations from Fig. 8 of the simulated total (cortical and ventricular) capacitance and resistance variations over the cardiac cycle obtained using FFT extrapolation.

As mentioned in Section 2.3.2, the *deformation data were extrapolated* to the brain surface using different methods (see Fig. 6 for visualizations of the *brain motion*). Fig. 9 shows – for the second electrode configuration and the different subjects – the *dC* and *dR* contributions from the cortical brain surface when the deformation data is extrapolated using the KNN, as well as the FFT method. Similarly, the *dC* and *dR* contribution from ventricular pulsation is shown in Fig. 10. The results obtained with the KNN and FFT methods are in general agreement, thus supporting the reliability of reconstruction methods.In Fig. 11, the (subject-averaged) *contributions from geometry pulsation and from perfusion-related dielectric properties* are distinguished. Furthermore, the signal parts originating from the cortical and ventricular CSF-brain interface parts are shown separately. Subject-averaged *dC* and *dR* for the *three electrode configurations* from Fig. 8 are shown in Fig. 12.

Subsequently, we compensated for overall impedance magnitude changes by determining appropriate offset and scaling factor (see Section 2.4) to investigate the *inter-subject variability*. The standard deviation of the scaling factors $f_{d,i}$ – a measure for the amplitude variability between subjects – is reported in Table 2 for the 2^nd^ electrode configuration and both reconstruction methods. The mean and standard deviations of the L2-norm of the difference between individual $d_{i}^{*}(t)$ and the subject-averaged curves (relative to the L2-norm of the average subject) are also provided in Table 2, as measures for the curve shape variability.

**Table 2:** Analysis of the variability between subject-specific *dC* and *dR* signals, shown for the 2^nd^ electrode configuration. To distinguish differences in signal magnitude and signal shape, scaling factors $f_{i}$ (*i*: subject) relative to the mean over all subjects were extracted (i.e., $d_{i}^{*}(t)=f_{d,i}\cdot d_{i}(t)+g_{d,i}$, where *d* can stand for *dC* or *dR*). The first row shows the standard deviation of the scaling factors of the eight subjects (i.e., magnitude variability). The mean and standard deviation of the L2-norm difference between scaled and shifted subject signals ($d_{i}^{*}(t)$) and the subject-averaged one, relative to the L2-norm of the average subject, are given in second and third row, respectively.

	**KNN**				**FFT**			
	**Cortical Interf.**		**Ventricular**		**Cortical Interf.**		**Ventricular**	
	dC	dR	dC	dR	dC	dR	dC	dR
**std.dev.**$f_{i}$	22%	25%	43%	37%	29%	30%	27%	33%
**mean**$\frac{||d_{i}^{*}(t)-d_{\mathrm{aver}}|{|}_{2}}{||d_{\mathrm{aver}}(t)|{|}_{2}}$	24%	23%	29%	27%	33%	32%	25%	21%
**std.dev.**$\frac{||d_{i}^{*}(t)-d_{\mathrm{aver}}|{|}_{2}}{||d_{\mathrm{aver}}(t)|{|}_{2}}$	6%	5%	14%	14%	9%	9%	12%	9%

The *uncertainties* associated with the discretization error and the dielectric tissue properties of the quantities-of-interest (*dC* and *dR*) were quantified for one subject and the 2^nd^ electrode configuration. Relative uncertainties in dB were determined for the peak-to-peak variation of *dC* and *dR*. The uncertainty quantification was performed using both the KNN and the FFT reconstructed deformation data, resulting in very similar uncertainty budgets. Here the values for the KNN data are reported. The results are shown in Table 3. To compute the combined numerical and dielectric uncertainty, the root sum of squares was used, resulting in an estimated 1.4dB and 1.6dB combined uncertainty for *dC* and *dR*. Additional uncertainty sources are discussed in Section 4.1.

**Table 3:** Uncertainty budget for the peak-to-peak magnitude of *dC* and *dR*, assuming log-normal uncertainty distributions with 10% standard deviation for the underlying dielectric tissue properties (shown based on the KNN reconstruction data; almost identical results are obtained using FFT reconstruction).

	**dC uncertainty (dB)**		**dR uncertainty (dB)**	
	σ	∊	σ	∊
**white matter**	0.18	0.11	0.30	0.07
**gray matter**	0.33	0.26	0.59	0.10
**CSF**	1.00	0.02	0.78	0.03
**dura**	0.07	0.01	0.16	0.03
**muscle**	0.01	0.04	0.37	0.06
**fat**	0.74	0.08	0.09	0.03
**blood**	0.05	0.00	0.03	0.04
**cortical bones**	0.03	0.04	0.02	0.04
**cancellous bones**	0.02	0.02	0.01	0.03
**galea and epicranial aponeurosis**	0.02	0.02	0.02	0.03
**skin**	0.00	0.24	0.00	0.04
				
**combined diel. uncertainty**	1.30	0.39	1.11	0.16
**numerical uncertainty**	0.13		1.2	
				
**combined uncertainty (**k=1**)**	1.4		1.6	

**Fig. 13:**
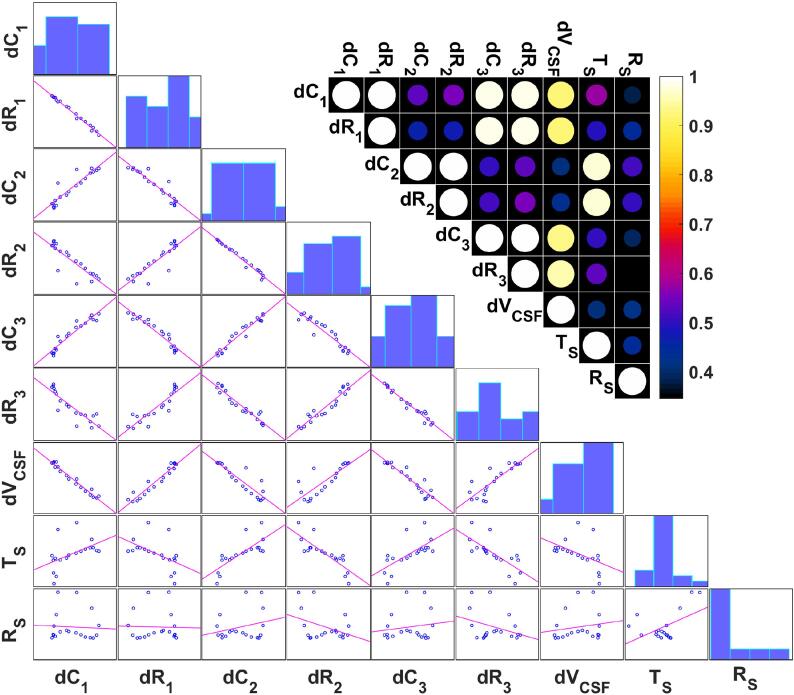
Correlation between the subject-averaged measurable signals and subject-averaged deformation data features using FFT-reconstructed data. ${\mathrm{dC}}_{1,2,3}$ and ${\mathrm{dR}}_{1,2,3}$: capacitance and resistance variation for 1^st^, 2^nd^, and 3^rd^ electrode configurations, ${\mathrm{dV}}_{\mathrm{CSF}}$: brain volume change, $T_{S}$: brain translation, and $R_{S}$: brain rotation. Each dot in the scatter plots corresponds to one time-point.

**Fig. 14:**
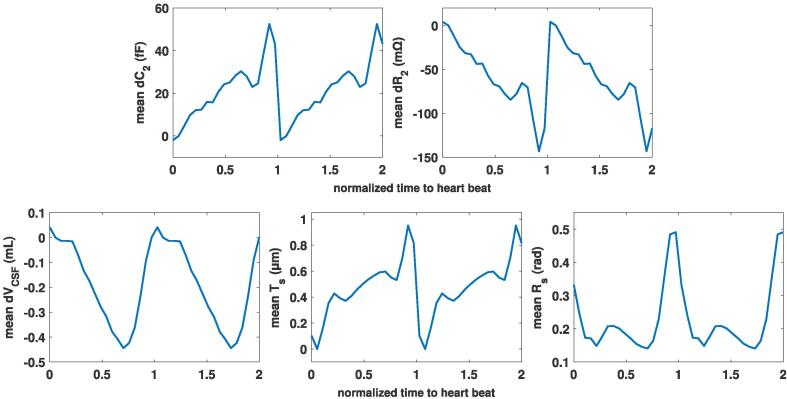
(Top) simulated measurable signals for the 2^nd^ electrode configuration using FFT reconstruction; (Bottom) ${\mathrm{dV}}_{\mathrm{CSF}}$: brain volume change, $T_{S}$: brain translation (surface averaged), and $R_{S}$: brain rotation (surface averaged).

**Fig. 15:**
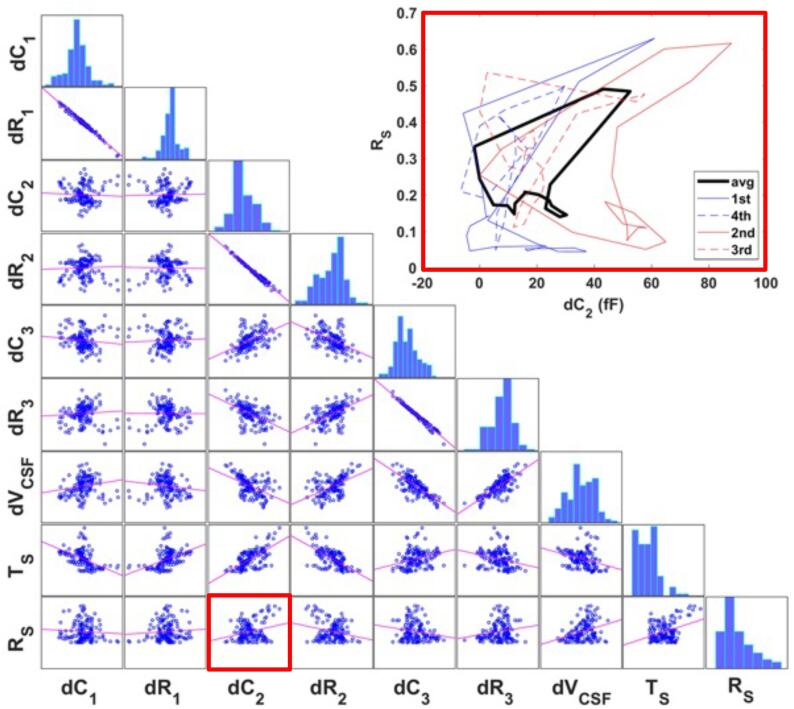
Correlation between the measurable signals and deformation data features using FFT reconstruction. ${\mathrm{dC}}_{1,2,3}$ and ${\mathrm{dR}}_{1,2,3}$: capacitance and resistance variation for 1^st^, 2^nd^ and 3^rd^ electrode configurations, ${\mathrm{dV}}_{\mathrm{CSF}}$: CSF volume change, $T_{S}$: total translation of the cortex, and $R_{S}$: total rotation of the cortex. Each dot in the scatter plots corresponds to one subject and one time-point. The nonlinear relationships (‘loops’; bold: mean over all subjects, dashed: illustrative individual subjects) between ${\mathrm{dC}}_{2}$ and $R_{S}$ are shown on the top right. In view of the multiple electrode pairs and the different deformation features, the loops should be viewed as being higher-dimensional (i.e., a vector of size $n_{t}\cdot (n_{f}+n_{s})\text{; }n_{t}$: number of time-points, $n_{f}$: number of features, $n_{s}$: number of signals).

Towards deriving an impedance-measurements-based CC surrogate, we investigated the *relationship between dC and dR and deformation data features*. Fig. 13 shows the relationship between measurable signals (average of eight subjects) and deformation data features, along with their linear correlation coefficients, for the three electrode configurations shown in Fig. 8 (see Fig. 14 for an illustration of a measurable signal and the features of the underlying deformation data). There is high linear correlation between *dC* and *dR* for a given electrode configuration (above 99%), which indicates that they carry the same information content. On average, the signals from the 1^st^ and 3^rd^ electrode configuration correlate well with the volume change ${\mathrm{dV}}_{\mathrm{CSF}}$ and the signal from the 2^nd^ one with the translation $T_{s}$, but Fig. 15 reveals that this relationship is not universal among subjects. It is evident that different electrode configurations are sensitive to different deformation features. Although there is low linear correlation between measurable signals and $R_{s}$, a closer look shows that there is a systematic nonlinear relationship between them (‘loops’, illustrated in Fig. 15). The loops result from the complexity of the brain pulsations, in which the back- and the forward path differ. Moreover, these loops are subject-specific, as evident in Fig. 15.

These observations motivated a further investigation in which *principal components* of these loops were related to principal components of the motion feature curves. For this, all measurable signals and deformation-feature-curves were normalized and combined in a single vector per subject, and a PCA was performed. The resulting principal components were projected to two sub-spaces (measurable signals and deformation data features) and renormalized.

**Fig. 16:**
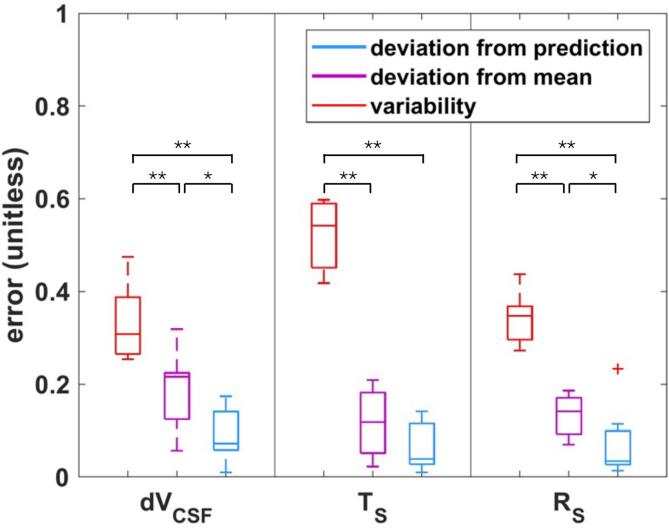
Box-plot of the deviations for all subjects of the measurement-signal-based volume change, translation, and rotation predictions from their real values. Those deviations are much smaller than the deviations of the different subjects from the mean, or than their variability (significance assessed using the Wilcoxon signed-rank test; ⋆:p-value<5%,⋆⋆:p-value<1%); ${\mathrm{dV}}_{\mathrm{CSF}}$: CSF volume change, $T_{S}$: brain translation (surface averaged), and $R_{S}$: brain rotation.

A relationship between the obtained measurement signal part of the principal components and the deformation feature part was established using ridge regression. Based on this relationship, it was possible to *estimate subject-specific deformation features from their measurement signals*, via projection into the principal component spaces and back. The deviation between the estimated deformation features ($x^{\prime }$) and the raw data (*x*) was quantified using the following metric:

(25) |x-x^|2∑ixi2∑ix^i2

and is reported in Fig. 16.

## 4 Discussion

In this study, we developed an accurate and efficient computational approach for solving dynamic EM problems with subtle dynamic variation. This approach was used to investigate the head impedance variation (*dC* and *dR*) over the cardiac cycle, as a potential intermediary to derive non-invasive CC surrogates. For this, computationally determined sensitivity maps were combined with experimentally obtained brain pulsation data.

Brain pulsation data are an important source of information, not only for early-stage diagnosis of multiple brain diseases, but also to advance our understanding of their pathophysiological basis. After processing the brain pulsation data, we visualized and studied one subject’s *transient brain motion* as a preliminary step toward understanding (healthy baseline) brain motion dynamics. The pulsation period was divided into six sub-intervals (see Fig. 6). By studying the streamlines of the motion-vector-field, six centers were identified that act as principal motion sources or sinks (shown as spheres in Fig. 6). These centers can slightly shift and invert their role over the duration of the pulsation period, as the brain moves back and forth (though not along the same trajectory). The bold arrows in Fig. 6 indicate prominent trajectories (streamline bundles) and permit to distinguish sources and sinks. The six centers are roughly arranged at the base of the skull (1; midbrain and cerebellum), the top (6), and the four corners of a horizontally positioned rectangle (2–5). They can be thought of as being arranged at the corners of an octahedron. Initially, there is a general, relatively uniform brain shift towards the top (moderate magnitude). Over the next three sub-intervals, the general motion is one in which the brain continues to shift towards but is stopped at the top, resulting in axial compression associated with stretching of the brain along an axis that is initially mainly anterior-posterior oriented, but subsequently tilts toward one of the rectangle-diagonals (left–right symmetry breaking). That stretching is associated with a related narrowing along the other (nearly perpendicular) diagonal. Subsequently, this concerted motion in the horizontal direction chaotically dissolves, leaving a tilting motion (up in front, down at the back). During the last sub-interval, the brain shifts down and the inverse of the stretching-thinning motion pattern in the horizontal plane manifests, restoring the original brain shape and position.

Towards deriving a non-invasive CC surrogate, a relationship was established that permits to *estimate subject-specific deformation features based on the measurable dC and dR.* Fig. 16 compares the error (according to Eq. (25)) from this subject-specific prediction (based on PCA) to the deviations between the subject-specific and the averaged feature curves and to the ‘variability’ between subjects (computed as the mean of the correlations with the other subjects). Clearly, the subject-specific predictions are capable of accounting for most of the inter-subject variability. This was confirmed using the Wilcoxon signed-rank test (see Section 2.6 and Fig. 16). Except for the comparison between the subject-specific translation curve predictions and their deviation from the subject-averaged curve, the superiority of the subject-specific predictions was confirmed in all cases (*p*-value<5%). Compared to the inter-subject variability, the superiority of the subject-specific predictions is always highly significant (*p*-value<1%).

Furthermore, the subject-averaged *dC* and *dR* for the three electrode configurations (see Fig. 12) reveal the potential of obtaining complementary information from their signals by optimizing multi-electrode placement. As evident in Fig. 8, the sensitivity is highest near the electrodes, which permits to spatially distinguish signal sources. The sensitivity maps can be used to *maximize the signal information content*, keeping in mind that the optimal locations can be disease-specific and can differ according to the diagnostic goal. The sensitivity maps also reveal that, while geometry pulsation and brain perfusion changes both contribute to the head impedance variation, the former dominates. This is further confirmed by the computed *dC* and *dR* signal contributions (Fig. 11).

### 4.1 Limitations

The approaches and methodologies developed in this study are promising and present an important step towards potential clinical application. However, the following study limitations must be considered and addressed in follow-up work.

*Modeling uncertainties.* While uncertainty contributions associated with dielectric properties and numerical errors were studied, other uncertainty contributions remain to be quantified. Particularly the reconstruction of the brain surface motion from the noisy image data and inter-subject anatomical variability (i.e., using registered MIDA instead of personalized head models) are expected to be major uncertainty contributors that are hard to assess in the absence of corresponding ground truth data. Also, instead of assuming 10% uncertainty for all dielectric properties, their real uncertainty/variability should be determined, e.g., based on the variability of the measurement data collected in Hasgall et al. (2018). Electrode contact is assumed to be perfect and no (capacitive) interface effects are considered. Other factors that deserve consideration are tissue heterogeneity and anisotropy, segmentation accuracy, and the non-enforced continuity of thin structures, such as the dura mater.

The implemented perfusion model assumes that brain volume changes can be attributed to corresponding perfusion changes that homogeneously affect brain dielectric properties based on a mixing rule. However, the 4D brain motion data reveals that it is not divergence free, and thus, perfusion changes are not homogeneously distributed. Yet, the deformation data quality does not permit to extract reliable maps of heterogeneous perfusion change.

Other signal sources, e.g., related to the pulsation of intra- and extra-cranial vasculature, or scalp tissues, have not been considered (see Andreas Spiegelberg et al., 2022, Boraschi et al., 2022 regarding experimental evidence for the intra-cranial origin of the signal). For blood vessels with small diameters that are fully embedded in a homogeneous tissue, the signal contributions from diametrically opposite vessel sides are expected to compensate, such that only large vessels on the brain surface or within the scalp/face are relevant.

*Disease conditions.* For this study, we only had access to deformation data from healthy volunteers. Additional data and investigations are needed to assess the impact of various disorders on head impedance variations and to conclude on their diagnostic value.

*Electrode configuration optimization.* In the present study, three specific electrode placements were considered. Electrode shapes and placements should be optimized to maximize the signal information content. The here established sensitivity map computation methodology can support this. Considering the varying dielectric dispersions of different tissues, it might even be valuable to vary the frequency, or to use multiple frequencies. The optimal configuration could depend on the targeted diagnostic information (e.g., different for different craniospinal diseases).

### 4.2 Future work

In addition to optimizing the electrode configuration and studying differences between healthy and disease conditions, the following aspects should be further investigated:

*Derivation of ICP/CC.* Compartmental models of intracranial fluid exchange, such as (Gehlen et al., 2017), might help relate brain pulsation information obtained through head impedance monitoring to ICP and/or CC. Alternative, heuristically derived relationships could be employed.

*Tomographic reconstruction.* By strongly increasing the number of electrodes, it might be possible to extend the presented methodology to a non-invasive, affordable, and accurate tomographic brain motion reconstruction method with high temporal resolution (well above that achievable with MRI). For that, a corresponding inverse problem needs to be solved. The ability of using computational modeling to identify electrode locations with complementary sensitivity fields will be of high value towards this goal.

## 5 Conclusion

In this study, we developed a reliable and efficient procedure for solving dynamic electrostatic problems where variations in the geometry and/or dielectric properties are much smaller than the static ones. The developed algorithm is general and applicable to any dynamically varying configuration that fulfills the mentioned criteria. It was verified against analytical and semi-analytical solutions in four different benchmarks.

The approach was developed for and applied to the challenge of relating measurable head impedance variations to underlying brain pulsation, as a surrogate for ICP and/or CC measurements, towards a non-invasive diagnostic biomarker for craniospinal diseases. Such a tool could be of high clinical value by enabling systematic patient screening, and by facilitating continuous monitoring of ICP and/or CC surrogates in the neurocritical care setting. By combining a large number of electrodes that differ in their sensitivity to different deformation contribution, a tomographic brain pulsation reconstruction method with high temporal resolution and accuracy might even be achievable.

The present computational study indicates that the measurable signal is dominated by the pulsatile displacement of the cortical brain surface, with minor contributions from the ventricular surfaces and from changes in brain perfusion. Different electrode setups result in complementary information, and the information content from the investigated three electrode pairs can be used to infer subject-specific brain pulsation and motion features with a high statistical significance.

In a forthcoming study, computational model predictions will be experimentally validated in healthy and diseased subjects using the recently developed device from Andreas Spiegelberg et al. (2022).

## Data and code availability statements

The Sim4Life simulation setups are available upon request from the corresponding author, provided the License Agreement for the MIDA anatomical head model has been signed and submitted to the FDA or the IT’IS Foundation (see itis.swiss/virtual-population/regional-human-models/mida-model/). The deformation image data is available from Prof. Jaco J.M. Zwanenburg upon reasonable request.

## CRediT authorship contribution statement

**Fariba Karimi:** Conceptualization, Methodology, Software, Validation, Formal analysis, Writing - original draft, Visualization. **Esra Neufeld:** Conceptualization, Methodology, Formal analysis, Writing - review & editing, Visualization, Supervision, Project administration, Funding acquisition. **Arya Fallahi:** Methodology, Formal analysis, Writing - review & editing, Supervision. **Andrea Boraschi:** Validation, Investigation, Writing - review & editing. **Jaco J.M. Zwanenburg:** Resources, Writing - review & editing. **Andreas Spiegelberg:** Conceptualization, Validation, Writing - review & editing. **Vartan Kurtcuoglu:** Conceptualization, Validation, Investigation, Writing - review & editing, Project administration, Funding acquisition. **Niels Kuster:** Resources, Writing - review & editing, Supervision.

## Declaration of Competing Interest

The authors declare the following financial interests/personal relationships which may be considered as potential competing interests: Andreas Spiegelberg is applicant and inventor of the patent application DE102018100697A1 and several dependent international applications. Vartan Kurtcuoglu is inventor and the University of Zurich applicant of the same patent applications.

## Data Availability

Data will be made available on request.
