# Supplementary material for: Theory for a non-invasive diagnostic biomarker for craniospinal diseases
Source: Neuroimage Clin. 2022 Dec 8;37:103280. doi: 10.1016/j.nicl.2022.103280 (PMC9763738; doi:10.1016/j.nicl.2022.103280)
Supplement: Supplementary data 1 [file mmc1.zip › mmc1/Supplementary Materials.pdf]

Supplementary Materials:

1. Semi-Analytical Benchmarks

As described in Section 2.2, analytical benchmark solutions of Eq. (1) are required to validate the reciprocity-theorem-based approach. This supplement derives such benchmarks with increasing asymmetry and complexity.

1.1. 1D benchmark

The following analytical solution of the Laplace equation for the 1D benchmark (see Fig. 3(a)) can easily be verified [1]:

$$\phi = \begin{cases} \dfrac{\tilde{\epsilon_2} x}{\tilde{\epsilon_2} p + \tilde{\epsilon_1}(1-p)} V_0 & x \leq p \\ \dfrac{\tilde{\epsilon_2} p + \tilde{\epsilon_1}(x-p)}{\tilde{\epsilon_2} p + \tilde{\epsilon_1}(1-p)} V_0 & p < x \leq 1 \end{cases} \tag{S1}$$

where p is the thickness of the dielectric slab with permittivity $\tilde{\epsilon_1}$. After computing the electric field $E = -\frac{d\phi}{dx}$, the electric charge density is obtained as follows:

$$Q = \int \tilde{\epsilon} \boldsymbol{E} \cdot \boldsymbol{dS} = \frac{\tilde{\epsilon_1}\tilde{\epsilon_2} V_0}{\tilde{\epsilon_2} p + \tilde{\epsilon_1}(1-p)} \tag{S2}$$

Q^* is obtained by substituting p with $p + dp$ and $\tilde{\epsilon_1}$ with $\tilde{\epsilon_1^*}$. As a result,

$$dQ_{direct} = Q^* - Q = -\tilde{\epsilon_2} V_0 \frac{\tilde{\epsilon_1}(\tilde{\epsilon_2} - \tilde{\epsilon_1^*})dp - \tilde{\epsilon_2} d\tilde{\epsilon_1} p}{(\tilde{\epsilon_2} p + \tilde{\epsilon_1}(1-p)) \left(\tilde{\epsilon_2}(p+dp) + \tilde{\epsilon_1^*}(1-p-dp)\right)}. \tag{S3}$$

On the other hand, the developed equation Eq. (21) leads to the following result for dQ:

$$dQ_{formula} = -\left(\frac{V_0}{(\tilde{\epsilon_2} p + \tilde{\epsilon_1}(1-p))^2}\right) \left(\tilde{\epsilon_1}\tilde{\epsilon_2}(\tilde{\epsilon_2} - \tilde{\epsilon_1})dp - \tilde{\epsilon_2}^2 d\tilde{\epsilon_1} p\right). \tag{S4}$$

The small differences between the analytical reference solution dQ_{direct} and estimations obtained using the reciprocity theorem-based approach $dQ_{formula}$ (shown in Fig. S1) support its validity

1.2. 2D symmetric benchmark

A solution to the 2D symmetric benchmark (see Fig. 3(b)) can be obtained starting from the general solution of the Laplace equation in cylindrical coordinates and enforcing potential and current conservation on $\rho = \rho_1$ and $\rho = \rho_2$ [1, 2]:

$$\phi = \begin{cases} \displaystyle\sum_{n=1}^{\infty} A_n \rho^n \sin(n\varphi) & \rho \leq \rho_1 \\ \displaystyle\sum_{n=1}^{\infty} \left(\rho^n + \frac{T-1}{T+1}\rho_1^{2n}\rho^{-n}\right) \frac{T+1}{2T} A_n \sin(n\varphi) & \rho_1 < \rho \leq \rho_2 \\ \displaystyle\sum_{n=1}^{\infty} \left(\rho_2^n + \frac{T-1}{T+1}\rho_1^{2n}\rho_2^{-n}\right) \frac{T+1}{2T} \rho_2^n A_n \rho^{-n} \sin(n\varphi) & \rho > \rho_2 \end{cases} \tag{S5}$$

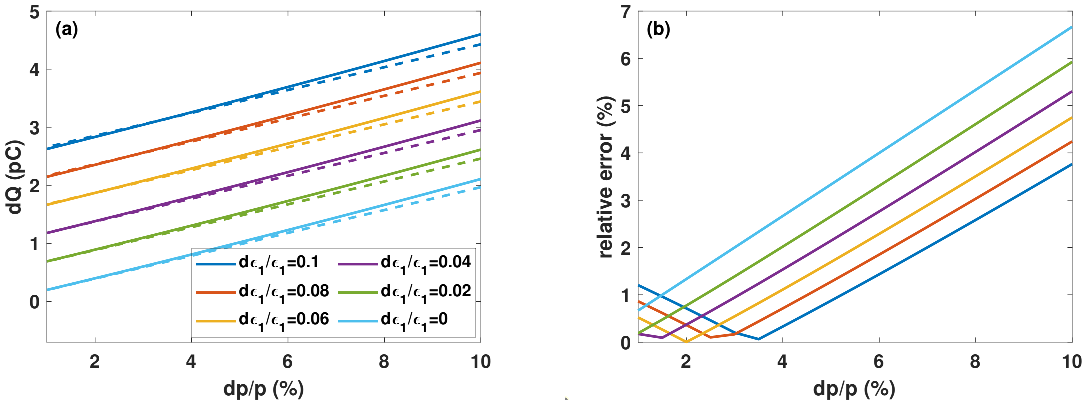

Figure S1: Verification of the reciprocity-theorem-based approach using the 1D benchmark with $\tilde{\epsilon_{r1}} = 2$, $\tilde{\epsilon_{r2}} = 10$, $p = 0.5$, and $V_0 = 1$. (a) dQ (electric charge variation) computed analytically (solid lines) along with dQ obtained using the approximation (dashed line), (b) relative error.

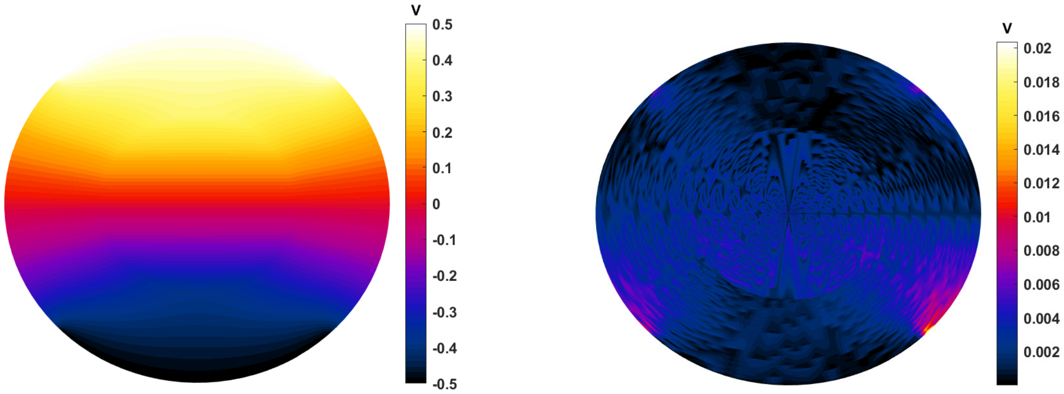

(a) Semi-analytical solution

(b) Absolute value of the difference between the semi-analytical and the numerical solutions

Figure S2: Electric potential distribution for the 2D symmetric benchmark (see Fig. 3(b)) with $\rho_1 = 0.5$, $\rho_2 = 1$, $\tilde{\epsilon_{r1}} = 2$, $\tilde{\epsilon_{r2}} = 10$, $\pi/4 \leq \varphi \leq 3\pi/4 \in \gamma$, $V_0 = 1$.

where $T = \frac{\tilde{\epsilon_2}}{\tilde{\epsilon_1}}$.

The coefficients A_n can be determined by imposing potential-continuity and current-conservation at $\rho = \rho_2$ through a point-matching algorithm. On the electrodes, the electric potential is known and elsewhere on the boundary the normal component of the displacement current must vanish as a result of the continuity of the normal component of the displacement current at $\rho = \rho_2$, which can be satisfied if and only if this quantity is equal to zero. We verified this semi-analytical benchmark solution against simulation results obtained using Sim4Life. Fig. S2 shows the electric field distribution and the difference between the semi-analytical and the numerical solutions. The small differences (always below 2 % of the dynamic range) increase the confidence in the semi-analytical solution.

After verifying the analytic solution, we used it to computed dQ and compared the results to those obtained using the reciprocity-theorem-based estimation (Eq. (21)). Fig. S3 shows that the relative error between dQ and dQ^* is less than 2.5 %.

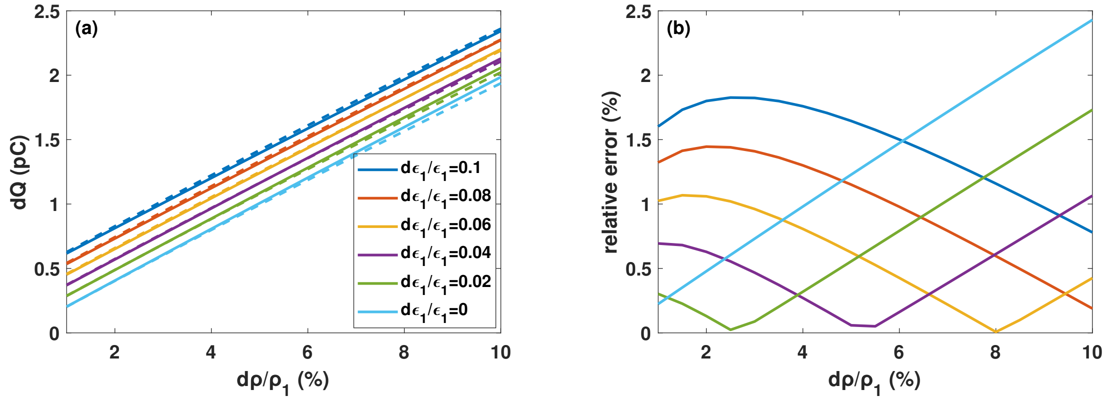

Figure S3: Verification results obtained for the 2D symmetric benchmark with $\rho_1 = 0.5$, $\rho_2 = 1$, $\tilde{\epsilon}_{r,1} = 2$, $\tilde{\epsilon}_{r,2} = 10$, $\varphi_- \leq \varphi \leq \varphi_+ \in \gamma$, and $V_0 = 1$, $\varphi_- = \pi/4$, and $\varphi_+ = 3\pi/4$. (a) dQ computed semi-analytically (solid lines) along with dQ computed using the approximation (dashed line), (b) relative error.

1.3. 2D asymmetric benchmark

To determine the electric potential in the '2D' asymmetric benchmark shown in Fig. 3(c) (asymmetric in the x, y-plane, translational symmetry along the z-axis), we first consider a dielectric cylinder of radius R with permittivity $\tilde{\epsilon_1}$ placed at distance b from a charged line wire with charge density q per unit length within a host medium with permittivity $\tilde{\epsilon_2}$ (Fig. S4). To solve this problem, we first find electric potential due to the wire in the absence of the dielectric cylinder. Using the general form of the Laplace equation for this problem and considering the following:

- the boundary condition is an even function and thus, the Fourier expansion of the electric potential does not contain $\sin(n\varphi)$.
- the electric potential should be finite when $\rho \to 0$
- for large ρ, the electric potential should be the same as for an infinite charged line on the z-axis.

we obtain (the origin of coordinate system is at the center of cylinder):

$$\phi_{wire} = \begin{cases} a_0 + \sum_{n=1}^{\infty} a_n \rho^n \cos(n\varphi) & \rho < b \\ b_0 ln(\rho) + \sum_{n=1}^{\infty} b_n \rho^{-n} \cos(n\varphi) & \rho > b \end{cases} \tag{S6}$$

The electric field and electric potential of a charged line with uniform linear charge density can be obtained as follows (Gauss' law):

$$\boldsymbol{E} = \frac{\lambda}{2\pi\epsilon\rho}\hat{\rho} \Rightarrow \phi = -\int_{\rho_A}^{\rho_B} \boldsymbol{E} \cdot dl = -\frac{\lambda}{2\pi\epsilon} \ln\left(\frac{\rho_B}{\rho_A}\right) \tag{S7}$$

where λ is the line charge density and ρ_A is the potential reference. By considering $\rho_A = 1$ and substituting Eq. (S7) in Eq. (S6), we get:

$$b_0 = -\frac{q}{2\pi\tilde{\epsilon_2}} \tag{S8}$$

Additionally, by imposing potential continuity at $\rho = b$:

$$\phi_{wire} = \begin{cases} -\dfrac{q}{2\pi\tilde{\epsilon_2}}\ln b + \displaystyle\sum_{n=1}^{\infty} c_n \left(\frac{\rho}{b}\right)^n \cos(n\varphi) & \rho < b \\ -\dfrac{q}{2\pi\tilde{\epsilon_2}}\ln \rho + \displaystyle\sum_{n=1}^{\infty} c_n \left(\frac{b}{\rho}\right)^n \cos(n\varphi) & \rho > b \end{cases} \tag{S9}$$

The remaining coefficient (c_n) are determined through Gauss' law at $\rho = b$. According to this law, we have:

$$\boldsymbol{n} \cdot (\boldsymbol{D_2} - \boldsymbol{D_1}) = \rho_s \tag{S10}$$

where ρ_s is the free surface charge density. So,

$$\frac{q}{2\pi b} + \tilde{\epsilon_2}\sum_{n=1}^{\infty} n c_n \frac{1}{b}\cos(n\varphi) + \tilde{\epsilon_2}\sum_{n=1}^{\infty} n c_n \frac{1}{b}\cos(n\varphi) = \frac{q}{b}\delta(\varphi) \tag{S11}$$

Multiplying Eq. (S11) by $\cos(m\varphi)$ and integrating over φ, we find that:

$$c_n = \frac{q}{2\pi n\tilde{\epsilon_2}} \tag{S12}$$

As a result:

$$\phi_{wire} = \begin{cases} \dfrac{q}{2\pi\tilde{\epsilon_2}}\left(-\ln b + \displaystyle\sum_{n=1}^{\infty} \frac{1}{n}\left(\frac{\rho}{b}\right)^n \cos(n\varphi)\right) & \rho < b \\ \dfrac{q}{2\pi\tilde{\epsilon_2}}\left(-\ln \rho + \displaystyle\sum_{n=1}^{\infty} \frac{1}{n}\left(\frac{b}{\rho}\right)^n \cos(n\varphi)\right) & \rho > b \end{cases} \tag{S13}$$

When the dielectric cylinder is present, it gives rise to $\phi_{cylinder}$ which can be expanded in a Fourier series of the form:

$$\phi_{cylinder} = \begin{cases} \displaystyle\sum_{n=1}^{\infty} C_n \left(\frac{\rho}{R}\right)^n \cos(n\varphi) & \rho < R \\ \displaystyle\sum_{n=1}^{\infty} C_n \left(\frac{R}{\rho}\right)^n \cos(n\varphi) & \rho > R \end{cases} \tag{S14}$$

which is derived from the general form of the Laplace equation in cylindrical coordinates by imposing a finite potential for $\rho \to 0$, asymptotic approximation of 0 at large ρ, and potential continuity. Imposing the continuity of normal component of current ($\tilde{\epsilon}(\boldsymbol{E}_{wire} + \boldsymbol{E}_{cylinder})$) at $\rho = R$, C_n is evaluated as follows:

$$C_n = -\frac{q}{2\pi n\tilde{\epsilon_2}}\frac{\tilde{\epsilon_1} - \tilde{\epsilon_2}}{\tilde{\epsilon_1} + \tilde{\epsilon_2}}\left(\frac{R}{b}\right)^n \tag{S15}$$

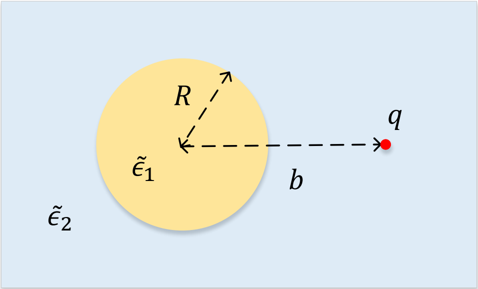

Figure S4: Geometry used as part of the derivation of the 2D asymmetric benchmark solution.

Substituting Eq. (S15) in Eq. (S14) leads to:

$$\phi_{cylinder} = \begin{cases} \dfrac{q}{2\pi\tilde{\epsilon}_2}\dfrac{\tilde{\epsilon}_1 - \tilde{\epsilon}_2}{\tilde{\epsilon}_1 + \tilde{\epsilon}_2}\displaystyle\sum_{n=1}^{\infty}\frac{1}{n}\left(\frac{\rho}{b}\right)^n \cos(n\varphi) & \rho < R \\ \dfrac{q}{2\pi\tilde{\epsilon}_2}\dfrac{\tilde{\epsilon}_1 - \tilde{\epsilon}_2}{\tilde{\epsilon}_1 + \tilde{\epsilon}_2}\displaystyle\sum_{n=1}^{\infty}\frac{1}{n}\left(\frac{R^2/b}{\rho}\right)^n \cos(n\varphi) & \rho > R \end{cases} \tag{S16}$$

By using Eq. (S14) and Eq. (S16), we can obtain for the potential of the geometry shown in Fig. S4:

$$\phi = \phi_{wire} + \phi_{cylinder} \tag{S17}$$

To solve the initial 2D asymmetric benchmark, the above formulation is used in the following procedure:

- consider each electrode as a set of infinite charged lines which carry q_i per unit length $(i = 1, 2, ..., M)$
- compute the electric potential at each point in the geometry using the superposition theorem
- find q_i such that: $\phi(\gamma_1) = V_0/2$ and $\phi(\gamma_2) = -V_0/2$ (by solving a system of linear equations)

We verified the proposed semi-analytical solution against the numerical one obtained using Sim4Life. The electric potential obtained semi-analytically and the difference compared to the numeric solution are shown in Fig. S5. In this example, neither the dielectric property distribution, nor the distance between the cylinder's center and the two electrodes are symmetric. The small difference (below 6 % of the dynamic range) enhance the confidence in the semi-analytical solution.

Following verification of the analytic solution, the direct dQ computation was compared to the results obtained using the newly developed formula (Eq. (21)). As shown in Fig. S6, the relative error between dQ and dQ^* is less than 5 %.

1.4. 3D benchmark

Solving the 3D benchmark (shown in Fig. 3(d)) by using the general solution of Laplace equation in spherical coordinate system and after enforcing the boundary conditions at

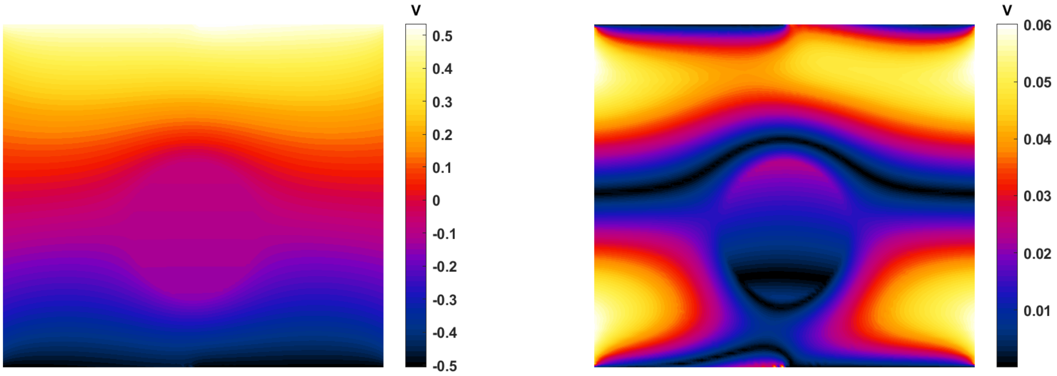

(a) Semi-analytical solution

(b) Absolute value of the difference between the semi-analytical and the numerical solution

Figure S5: Electric potential distribution in the 2D asymmetric benchmark with $a = 1.5$, $V_0 = 1$, $\tilde{\epsilon_{r1}} = 10$, $\tilde{\epsilon_{r2}} = 1$, $d_1 = 1.5$, and $d_2 = 1$. The geometry of this benchmark is shown in Fig. 3(c)

.

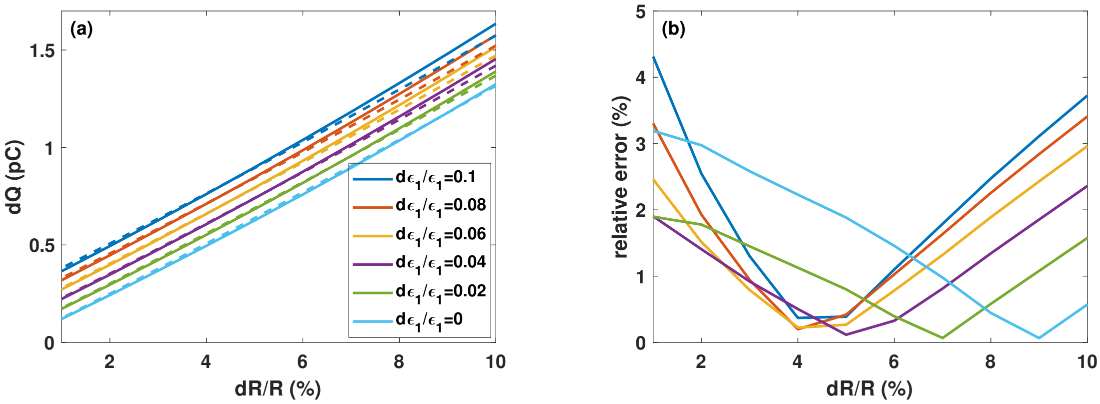

Figure S6: Verification results obtained for the 2D asymmetric benchmark with $a = 1.5$, $V_0 = 1$, $\tilde{\epsilon_{r,1}} = 10$, $\tilde{\epsilon_{r,2}} = 2$, and $d_1 = d_2 = 1$. (a) dQ computed using the analytical solution (solid lines) along with dQ computed using the approximation (dashed line), (b) relative error.

$r = r_1$ and $r = r_2$, we obtain for the electric potential distribution: [1, 2]:

$$\phi = \begin{cases} \displaystyle\sum_{n=0}^{\infty} A_n r^n P_n^0(\cos\theta) & r \leq r_1 \\ \displaystyle\sum_{n=1}^{\infty} A_n \left(r^n + \frac{T-1}{1+T\frac{n+1}{n}} r_1^{2n+1} r^{-(n+1)} \right) \frac{1+T\frac{n+1}{n}}{\frac{2n+1}{n}T} P_n^0(\cos\theta) & r_1 < r \leq r_2 \\ \displaystyle\sum_{n=1}^{\infty} A_n r_2^{n+1} \left(r_2^n + \frac{T-1}{1+T\frac{n+1}{n}} r_1^{2n+1} r_2^{-(n+1)} \right) \frac{1+T\frac{n+1}{n}}{\frac{2n+1}{n}T} r^{-(n+1)} P_n^0(\cos\theta) & r > r_2 \end{cases} \tag{S18}$$

where $P_n^0(.)$ is the Legendre function of the first kind and $T = \frac{\tilde{\epsilon_2}}{\tilde{\epsilon_1}}$.

As for the 2D symmetric benchmark, continuity of the normal component of the displacement current at $r = r_2$ on the parts of the surface with no electrode is only achieved if

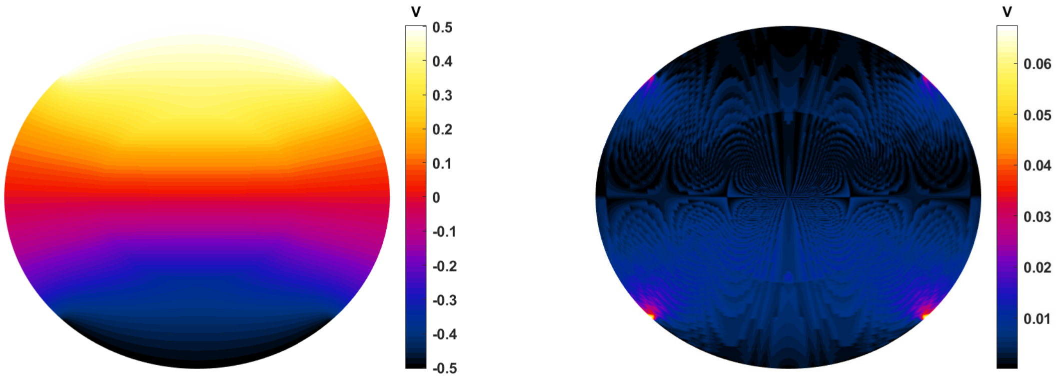

(a) Semi-analytical solution

(b) Absolute value of the difference between the analytical and the numerical solution

Figure S7: Electric potential distribution in the 3D benchmark, shown in the xz-plane, with $r_1 = 0.5$, $r_2 = 1$, $\epsilon\tilde{r}_1 = 2$, $\epsilon\tilde{r}_2 = 10$, $\pi/4 \leq \varphi \leq 3\pi/4 \in \gamma$, and $V_0 = 1$. The geometry of this benchmark is shown in Fig. 3(d)
.

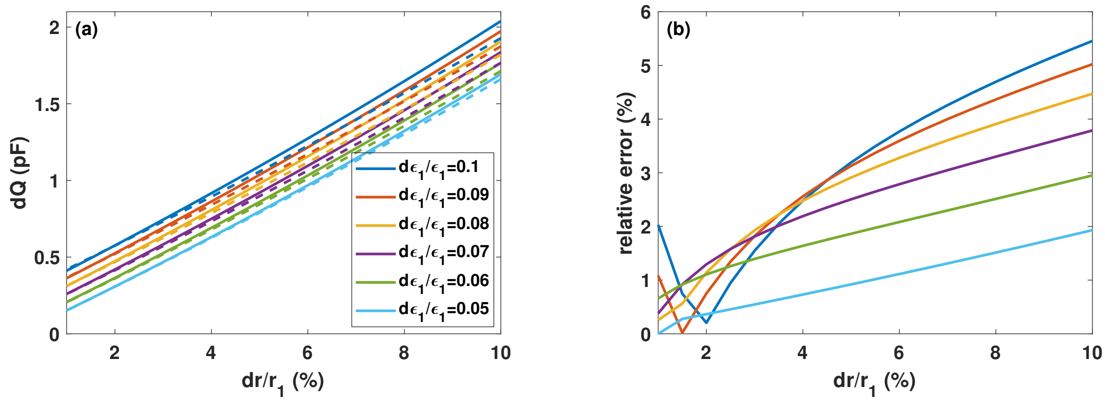

Figure S8: Verification results obtained for the 3D benchmark with $r_1 = 0.5$, $r_2 = 1$, $\epsilon_{\tilde{r},1} = 2$, $\epsilon_{\tilde{r},2} = 10$, $\pi/4 \leq \varphi \leq 3\pi/4 \in \gamma$, and $V_0 = 1$. (a) dQ computed analytically (solid lines) along with dQ computed using the approximation (dashed line), (b) relative error.

the it vanishes there. Then, the only unknown coefficient, A_n, can be computed using the boundary condition at $r = r_2$ by solving a system of linear equations for A_n. As for the previous benchmarks, we verified the proposed semi-analytical solution for each benchmark against the numerical one from Sim4Life. Fig. S7 shows the distribution of the semi-analytic electric potential in the xz-plane and also the difference between the semi-analytic and the numeric solutions.The small difference (below 2 % of the dynamic range mostly; up to 7 % at the electrode edges enhance the confidence in the semi-analytical solution.

As for the previous benchmarks, we compared dQ from the semi-analytical reference solution to the estimation obtained using the developed formula (Eq. (21)). Fig. S8 illustrates that the relative error between dQ and dQ^* is less than 6 %.

2. Supplementary Figures

Figure S9: Animated 3D streamlines of the brain motion associated with sub-interval (II) in the cardiac cycle (see Fig. 6).

Figure S10: Animated variant of Fig. 7 showing how the transient brain pulsation data from 4D MRI is coupled with the sensitivity maps extracted from MIDA-based EM simulations to produce the dC and dR signal contributions.

References

[1] David Keun Cheng. *Field and wave electromagnetics.* Pearson Education India, 1989.

[2] Fariba Karimi, Ahmadreza Attarpour, Rassoul Amirfattahi, and Abolghasem Zeidaabadi Nezhad. Computational analysis of non-invasive deep brain stimulation based on interfering electric fields. *Physics in Medicine & Biology*, 64(23):235010, 2019. Publisher: IOP Publishing.
