# Supplementary figures and images for: Theory for a non-invasive diagnostic biomarker for craniospinal diseases
Source: Neuroimage Clin. 2022 Dec 8;37:103280. doi: 10.1016/j.nicl.2022.103280 (PMC9763738; doi:10.1016/j.nicl.2022.103280)

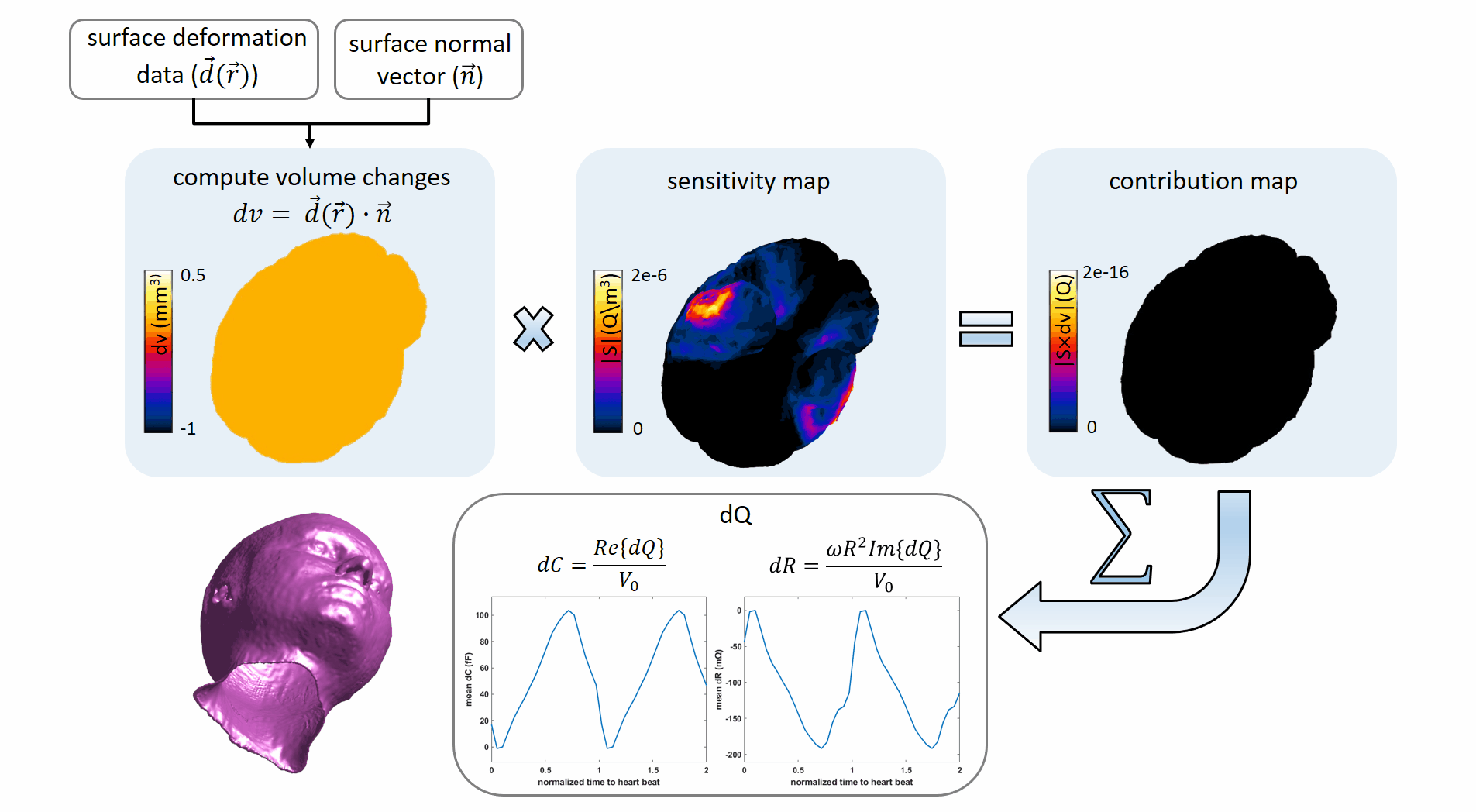

Supplement: Supplementary data 1 [file mmc1.zip › mmc1/Sup_Fig_10.gif]
